# A common polymorphism in the Intelectin-1 gene influences mucus plugging in severe asthma

**DOI:** 10.1038/s41467-024-48034-5

**Published:** 2024-05-09

**Authors:** Jamie L. Everman, Satria P. Sajuthi, Maude A. Liegeois, Nathan D. Jackson, Erik H. Collet, Michael C. Peters, Maurizio Chioccioli, Camille M. Moore, Bhavika B. Patel, Nathan Dyjack, Roger Powell, Cydney Rios, Michael T. Montgomery, Celeste Eng, Jennifer R. Elhawary, Angel C. Y. Mak, Donglei Hu, Scott Huntsman, Sandra Salazar, Luigi Feriani, Ana Fairbanks-Mahnke, Gianna L. Zinnen, Cole R. Michel, Joe Gomez, Xing Zhang, Vivian Medina, Hong Wei Chu, Pietro Cicuta, Erin D. Gordon, Pamela Zeitlin, Victor E. Ortega, Nichole Reisdorph, Eleanor M. Dunican, Monica Tang, Brett M. Elicker, Travis S. Henry, Eugene R. Bleecker, Mario Castro, Serpil C. Erzurum, Elliot Israel, Bruce D. Levy, David T. Mauger, Deborah A. Meyers, Kaharu Sumino, David S. Gierada, Annette T. Hastie, Wendy C. Moore, Loren C. Denlinger, Nizar N. Jarjour, Mark L. Schiebler, Sally E. Wenzel, Prescott G. Woodruff, Jose Rodriguez-Santana, Chad G. Pearson, Esteban G. Burchard, John V. Fahy, Max A. Seibold

**Affiliations:** 1 Center for Genes, Environment, and Health, National Jewish Health, Denver, CO USA; 2 Department of Medicine, University of California-San Francisco, San Francisco, CA USA; 3 Department of Cell and Developmental Biology, University of Colorado—Anschutz Medical Campus, Aurora, CO USA; 4 Division of Pulmonary, Critical Care, Allergy, and Sleep Medicine, Department of Medicine, University of California–San Francisco, San Francisco, CA USA; 5 Department of Genetics and Comparative Medicine, Yale University School of Medicine, New Haven, CT USA; 6 Department of Pharmaceutical Sciences, University of Colorado—Anschutz Medical Campus, Aurora, CO USA; 7 Biological and Soft Systems Sector, Cavendish Laboratory, University of Cambridge, Cambridge, UK; 8 Centro de Neumología Pediatrica, San Juan, PR USA; 9 Department of Medicine, National Jewish Health, Denver, CO USA; 10 Department of Pediatrics, National Jewish Health, Denver, CO USA; 11 Mayo Clinic, Phoenix, AZ USA; 12 School of Medicine, St. Vincent’s University Hospital, University College Dublin, Dublin, Ireland; 13 University of California-San Francisco, San Francisco, CA USA; 14 Duke University, Durham, NC USA; 15 Department of Internal Medicine, Mayo Clinic, Phoenix, AZ USA; 16 University of Kansas Medical Center, Kansas City, KS USA; 17 Cleveland Clinic, Cleveland, OH USA; 18 Harvard University, Cambridge, MA USA; 19 Brigham and Women’s Hospital and Harvard University, Cambridge, MA USA; 20 Pennsylvania State University, Hershey, PA USA; 21 Department of Medicine, Washington University School of Medicine, St. Louis, MO USA; 22 Washington University, St. Louis, MO USA; 23 Wake Forest University School of Medicine, Department of Internal Medicine, Section on Pulmonary, Critical Care, Allergy and Immunologic Diseases, Winston Salem, NC USA; 24 University of Wisconsin-Madison, Madison, WI USA; 25 University of Pittsburgh, Pittsburgh, PA USA; 26 Division of Pulmonary Sciences and Critical Care Medicine, Department of Medicine, University of Colorado—Anschutz Medical Campus, Aurora, CO USA

**Keywords:** Gene expression profiling, Transcriptomics, Asthma, Mechanisms of disease

## Abstract

By incompletely understood mechanisms, type 2 (T2) inflammation present in the airways of severe asthmatics drives the formation of pathologic mucus which leads to airway mucus plugging. Here we investigate the molecular role and clinical significance of intelectin-1 (ITLN-1) in the development of pathologic airway mucus in asthma. Through analyses of human airway epithelial cells we find that *ITLN1* gene expression is highly induced by interleukin-13 (IL-13) in a subset of metaplastic MUC5AC^+^ mucus secretory cells, and that ITLN-1 protein is a secreted component of IL-13-induced mucus. Additionally, we find ITLN-1 protein binds the C-terminus of the MUC5AC mucin and that its deletion in airway epithelial cells partially reverses IL-13-induced mucostasis. Through analysis of nasal airway epithelial brushings, we find that *ITLN1* is highly expressed in T2-high asthmatics, when compared to T2-low children. Furthermore, we demonstrate that both ITLN-1 gene expression and protein levels are significantly reduced by a common genetic variant that is associated with protection from the formation of mucus plugs in T2-high asthma. This work identifies an important biomarker and targetable pathways for the treatment of mucus obstruction in asthma.

## Abstract (web summary)

Type 2 inflammation drives the formation of pathologic mucus in patients with asthma. Here, authors reveal a role for intelectin-1 in IL-13-induced mucus properties, and that an *ITLN1* eQTL is associated with protection from the formation of mucus plugs in T2-high asthma.

## Introduction

Airway molecular characterization (endotyping) studies have revealed significant pathobiological heterogeneity among patients with asthma^1,2^. The most prevalent asthma endotype involves inflammation of the airways driven by type 2 (T2) cytokines, including interleukins 4, 5, and 13 (IL-4, IL-5, IL-13)^3–7^. Airway T2 inflammation is associated with more severe airflow obstruction, which may result from pathologic mucus production, leading to airway plugging. Indeed, computed tomography (CT) lung scans from patients with asthma show a high prevalence of airway mucus plugs, which correlate strongly with measures of airway T2 inflammation and airflow obstruction^8^. Supporting the involvement of T2 inflammation in mucus dysfunction, in vitro analyses of human airway epithelial cell cultures have uncovered that IL-13 triggers mucus hypersecretion and the metaplastic generation of mucus secretory cells, which produce a pathologic mucus that arrests mucociliary movement^5,9^.

The mechanisms underlying the formation of pathologic, T2 inflammation-induced airway mucus are unclear but may involve changes to the protein composition of the mucus. Clinical studies show that the normal predominance of MUC5B mucin in healthy airway mucus is shifted towards higher expression of MUC5AC in asthma^10^. Supporting a pathologic role for MUC5AC, a recent study found that MUC5AC protein is poorly extruded from the epithelium and can tether the mucus gel to the epithelium, limiting its mucociliary movement^9^. The potential for other protein components of mucus to be involved in T2-related mucus pathology was suggested by our recent study finding that IL-13-stimulated mucociliary airway epithelial cultures secrete a unique repertoire of proteins^5^. Among these proteins was intelectin-1 (ITLN-1), a lectin that can bind galactosyl glycans expressed by microbes but does not bind to mammalian glycans^11,12^. Importantly, ITLN-1 has also been reported to be a prominent protein constituent of mucus plugs in fatal asthma, although its role in airway mucus pathology is unknown^13^. Notably, a genetic variant at the *ITLN1* locus is associated with both Crohn’s disease and asthma, suggesting a role in inflammatory mucosal diseases^14–16^.

Here, by using scRNA-seq and CRISPR gene editing of human mucociliary airway epithelia, we define the role of ITLN-1 protein in IL-13-induced mucostasis, and demonstrate the ability of ITLN-1 to bind airway mucins via non-lectin-based electrostatic interactions. In asthma cohort studies in children and adults, we define the genetic and inflammatory regulators of *ITLN1* expression, and we show how functional genetic variation in the *ITLN1* gene influences susceptibility to airway mucus plugging.

## Results

### IL-13 mucus metaplastic epithelia produce mucus secretions containing ITLN-1

**Fig. 1:**
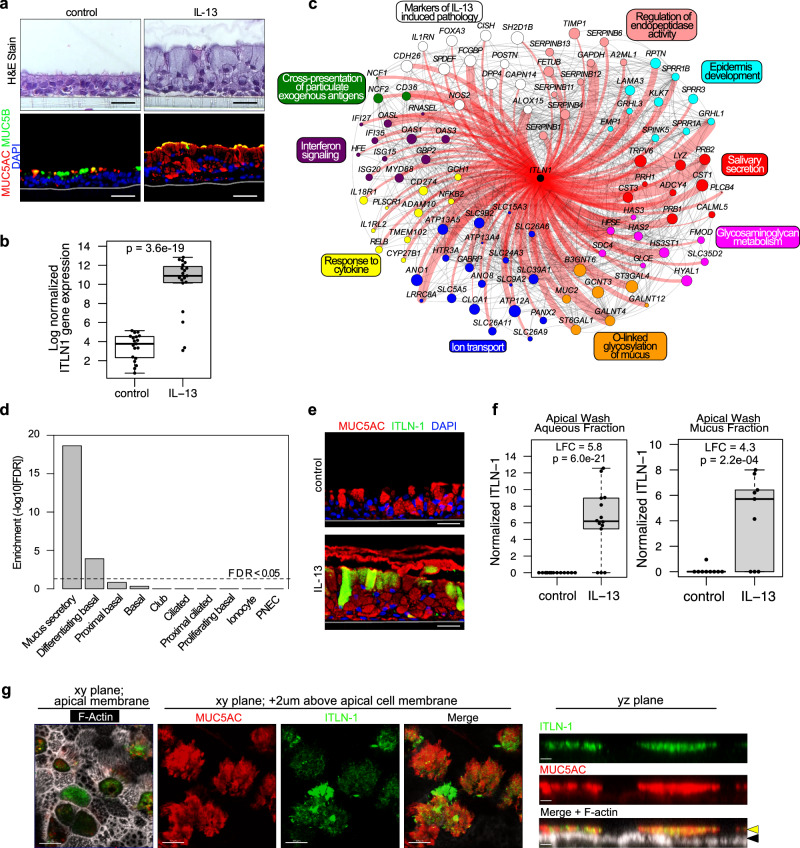
ITLN-1 is an IL-13-induced component of the airway epithelium. **a** Chronic IL-13 stimulation model of airway epithelial T2 inflammation as illustrated by H&E staining and immunofluorescent (IF) labeling of MUC5AC (red), MUC5B (green), and nuclei (blue); images are representative of 8 paired donors analyzed; scale bar 30 µM. **b** Box plot of normalized *ITLN1* gene expression in baseline (control) and IL-13-treated ALI cultures (*n* = 19 donors). FDR-adjusted (Benjamini-Hochberg method) two-sided *p* value is based on a paired exact test (edgeR). Box centers = median, upper and lower box bounds = 1st and 3rd quartiles, whiskers extend from these bounds up to 1.5 × IQR (inter-quartile range), and data beyond whiskers are plotted as points. **c** Connectivity among genes within a co-expression network that includes *ITLN1*. Genes shown are those annotated for the enriched functional terms and pathways indicated. Genes with direct connections to *ITLN1* are highlighted in red and edge width indicates strength of connectivity. Node color denotes functional annotation, and node size increases with eigengene-based connectivity to the network. **d** Enrichment of *ITLN1* co-expression network genes in mucus secretory cells, compared to all other defined airway epithelial lung cell populations identified by scRNA-seq. FDR-adjusted two-sided p-values are based on a one-sided hypergeometric test. **e** Immunofluorescent labeling shows IL-13-induced expression of ITLN-1 in a subset of MUC5AC^+^ secretory cells; MUC5AC (red), ITLN-1 (green), nuclei (blue); Images are representative of 8 paired donors analyzed; scale bar 30 µM. **f** Boxplots of normalized ITLN-1 peptides measured in apical ALI secretions following control and chronic IL-13 stimulation (aqueous fraction *n* = 14 donors, mucus fraction *n* = 9 donors). FDR-adjusted two-sided *p* values are based on a paired exact test (edgeR). LFC = log fold change. Box plots as defined in **b**. **g** Confocal imaging analysis of mucociliary ALI cultures stimulated with IL-13 illustrating ITLN-1 presence within the airway mucus on the apical side of mucociliary epithelium; MUC5AC (red), ITLN-1 (green), F-actin (white); black arrows—apical cell membrane, yellow arrows—mucus layer; XY image scale bar 10 µm; YZ image scale bar 2.5 µm; images are representative of ALI cultures labeled from *n* = 3 HBEC donors. Source data are provided as a Source Data file.

To investigate airway epithelial regulation and function of ITLN-1 we generated mucociliary airway epithelial air-liquid interface (ALI) cultures from primary human bronchial epithelial basal stem cells (HBEC) of 13 asthmatic and 6 healthy control donors. To model the effects of airway type 2 inflammation on ITLN-1, paired cultures were chronically stimulated (culture days 11-21) with interleukin 13 (IL-13), which resulted in mucus metaplasia (Fig. 1a). RNA-seq expression profiling revealed *ITLN1* was poorly expressed in control cultures, but was upregulated 170-fold among IL-13 treated cultures (*p* = 3.6e-19; Fig. 1b). No differences in either baseline or IL-13-induced *ITLN1* expression were observed between cultures derived from healthy versus asthmatic donors (SFig. 1a). To better understand the cellular and mechanistic context of *ITLN1* induction by IL-13, we performed co-expression network analysis on all expressed, variable genes, identifying 18 networks broadly representing different epithelial cellular functions and molecular pathways. *ITLN1* was part of a 598 gene network (Supplementary Data 1), which included canonical T2 inflammation markers (*POSTN*, *CST1*, *CDH26*), markers of mucus secretory cells (*FCGBP*, *CLCA1*, *DPP4*), and established transcription factor drivers of IL-13-induced mucus metaplasia (*SPDEF* and *FOXA3*; Fig. 1c). Formal enrichment analysis of the *ITLN1* network genes using GO, KEGG, and Reactome databases identified pathways involved in mucin formation and mucus secretion, including ion transport and O-linked glycosylation of mucins (Fig. 1c). These *ITLN1* network genes were strongly enriched (FDR 2.3e-19) for marker genes of mucus secretory cells based on human lung scRNA-seq data (Fig. 1d)^17^. Morever, we utilized CIBERSORTx to perform cell type deconvolution of the samples and then correlated *ITLN1* network expression with the cell type proportions, finding *ITLN1* network expression was most strongly, positively correlated with the proportion of the mucus secretory 2 (r = 0.74) and early secretory cells (*r* = 0.73) (SFig. 1d).

We next evaluated whether ITLN-1 protein was localized to mucus secretory cells, as suggested by our gene expression network analysis. Strikingly, we found control cultures, which predominantly express MUC5B protein, had no observable ITLN-1 protein, despite expressing a low, but measurable level of *ITLN1* mRNA. In contrast, we found prominent cytoplasmic ITLN-1 protein was present in IL-13 cultures, specifically among a subset of MUC5AC^+^ mucus secretory cells (Fig. 1e). Mass spectrometry-based proteomic analysis of culture-derived apical secretions detected ITLN-1 peptides in both the aqueous and mucus secretions of the IL-13-treated cultures, while ITLN-1 peptides were not detected in control culture secretions (Fig. 1f). ITLN-1 protein secretion did not differ significantly between cultures derived from healthy versus asthmatic donors (SFig. 1b, c). We confirmed the presence of ITLN-1 in airway mucus using confocal imaging of immunofluorescent (IF)-labeled IL-13 cultures, where we found ITLN-1 protein interspersed throughout the epithelial mucus layer (Fig. 1g). Together, our results demonstrate that ITLN-1 gene and protein expression are specifically induced by IL-13-driven mucus metaplastic airway epithelia, with ITLN-1 protein being apically secreted and integrated into mucus.

### Loss of ITLN-1 protein partially restores IL-13-mediated mucostasis

**Fig. 2:**
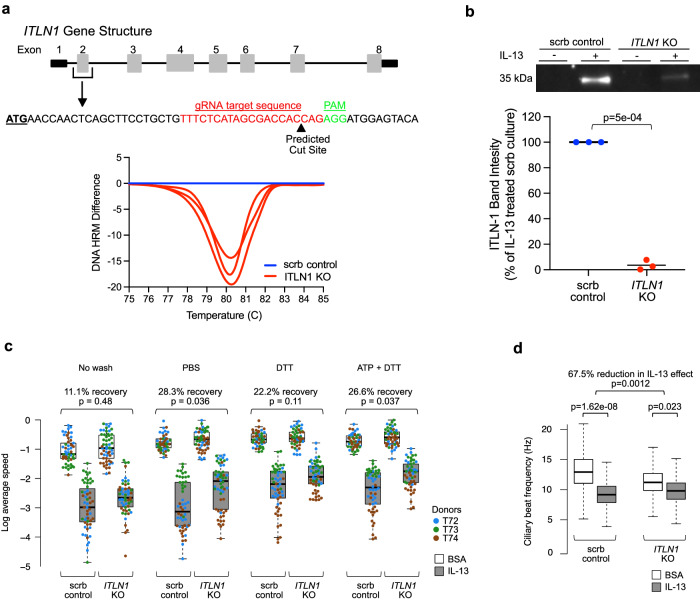
ITLN-1 contributes to IL-13-mediated decrease in epithelial mucociliary function. **a** Schematic of the gene structure of *ITLN1*, the targeted CRISPR-Cas9 editing site, and the resultant DNA editing as determined by high-resolution melt curve analysis. The plot (bottom) shows differences in melt temperature profiles from pooled ALI inserts (*n* = 3/condition) for *ITLN1* KO (red) samples from each donor assayed by HRM analysis. The scramble control DNA melt temperature profile (blue) was set as the reference for each donor (*n* = 3 donors). **b** Western blot quantitation of ITLN-1 (35 kDa) in apical washes collected from mock or IL-13-stimulated mucociliary ALI cultures differentiated from control edited (scrb; blue) and *ITLN1* KO basal cells (red); Western blot image is representative of all measured data, and band intensity plot reflects data from conditions from all edited donors (*n* = 3); Two-sided *p* values calculated using paired Student’s *t* test. **c** Box plots showing the log of average particle speed within control edited (scrb) and *ITLN1* KO mucociliary cultures that were mock- (BSA, white boxes) or IL-13-stimulated (gray boxes), with replicate experiments carried out using no wash, PBS wash, PBS-DTT wash, and ATP + PBS-DTT wash regimes. Data values represent the log of average speed of all measured particles within each video from the MCM assays, where there are an average of *n* = 18 videos for each of the 16 total conditions (2 gene-edited statuses × 2 treatments × 4 washes) per donor. Videos were captured across 6–7 fields of view across the culture to account for variation across the culture, measured across *n* = 3 individual ALI inserts from each of the edited donors (*n* = 3)—with each donor identified by a different color in the plot). The exact sample sizes per box, left to right, are 54, 55, 56, 55, 55, 54, 58, 58, 55, 55, 55, 56, 56, 54, 55, 58. Above plots are given the estimated percent recovery (and associated *p* values) of particle speed in IL-13-stimulated cultures relative to BSA cultures when in an *ITLN1* KO epithelium compared to the control (scrb) epithelium. Two-sided *p* values are based on linear mixed model *t* tests, with random effects for donor and insert, and use Satterthwaite approximation of degrees of freedom. Box centers = median, upper and lower box bounds = 1st and 3rd quartiles, whiskers extend from these bounds up to 1.5 × IQR (inter-quartile range). All data points are overlain. **d** Box plots of ciliary beat frequency (CBF) measured on control (scrb) and *ITLN1* KO mucociliary ALI cultures from triplicate culture inserts and 3 donors following mock- and IL-13-stimulation. Each box plot, as defined in **c**, represents all datapoints collected from all videos captured across donors and inserts for each condition (*n* = 30,899, 24,786, 25,533, and 24,964, from left to right), but illustration of outliers was excluded for visualization purposes. Two-sided *p* values are based on linear mixed model t-tests with random effects for donor and insert and use Satterthwaite approximation of degrees of freedom. Source data are provided as a Source Data file.

Given the differential presence of ITLN-1 in the mucus of control versus IL-13 metaplastic epithelial cultures, we hypothesized that IL-13-induced mucostasis could be mediated by ITLN-1. Generation of ITLN-1 knockout tracheal airway epithelial basal cells through CRISPR-Cas9 targeting of the *ITLN1* gene resulted in highly efficient editing of genomic DNA at the *ITLN1* target site as measured by high-resolution melt curve analysis (Fig. 2a). Additionally, high indel rates of 76%, 79%, and 82% at the *ITLN1* target site across the 3 edited donors were achieved, as measured by Inference of CRISPR Edits (ICE) analysis. These *ITLN1*-targeted and mock-targeted basal cells were used to generate mucociliary ALI cultures for functional evaluation. *ITLN1*-targeted cultures treated with IL-13 exhibited a 96.5% loss of apically secreted ITLN-1 protein as compared to the IL-13-treated mock-targeted cultures (Fig. 2b). *ITLN1* KO was not associated with changes in the expression of secretory genes known to be modified by IL-13, including *MUC5AC*, *MUC5B*, or *SCGB1A1* (SFig. 2a–c). Moreover, IL-13-induced secretion of MUC5AC was not altered by *ITLN1* KO (SFig. 2d), indicating that similar mucociliary differentiation and IL-13 responses were achieved by the *ITLN1* KO versus mock-edited mucociliary cultures. We next assessed mucociliary movement (MCM) among mock-targeted and *ITLN1* KO cultures, with and without IL-13 treatment, using video-based fluorescent bead tracking. These MCM measurements were performed on cultures prior to washing the mucus layer, after a PBS wash (to disrupt the aqueous mucus components), following a PBS-DTT wash (to disrupt mucus disulfide protein bonds), and after ATP stimulation (to induce release of intracellular mucin granules) followed by PBS-DTT wash. As expected, among mock-edited cultures, IL-13 treatment decreased particle speed in unwashed (2.80-fold), PBS washed (3.80-fold), PBS-DTT washed (3.61-fold), and ATP + PBS-DTT washed cultures (3.36-fold) (Fig. 2c), with similar results observed for particle displacement (SFig. 3a). However, among *ITLN1* KO cultures we found that 11% (unwashed, *p* = 0.48), 28% (PBS washed, *p* = 0.036), 22% (PBS-DTT washed, p = 0.11), and 27% (ATP + PBS-DTT washed, *p* = 0.037) of this IL-13-induced reduction in particle speed was restored (Fig. 2c). Similar results were also found for particle displacement (SFig. 3a). Direct evaluation of cilia movement through the measurement of ciliary beat frequency (CBF) in these cultures demonstrated that IL-13 treatment resulted in a 31% reduction in CBF in control cultures (*p* value = 1.62e-08; Fig. 2d, SFig. 4a). In contrast, we found only a 12% reduction in CBF induced by IL-13 (*p* = 0.023) among the *ITLN1* KO mucociliary cultures, constituting a 67.5% reduction in the effect of IL-13 in ITLN-1 KO cultures (*p* = 0.0012). An independent regeneration of mucociliary epithelia from these donor *ITLN1* KO and mock-edited basal cells, followed by MCM (no ATP stimulation performed) and CBF assays achieved similar results (SFig. 3b, c. SFig. 4b, c). These data suggest that apically secreted ITLN-1 protein plays a consequential role in the mucostatic effects that occur in the airway epithelium under IL-13-induced inflammation.

### ITLN-1 binds MUC5AC through electrostatic interactions

**Fig. 3:**
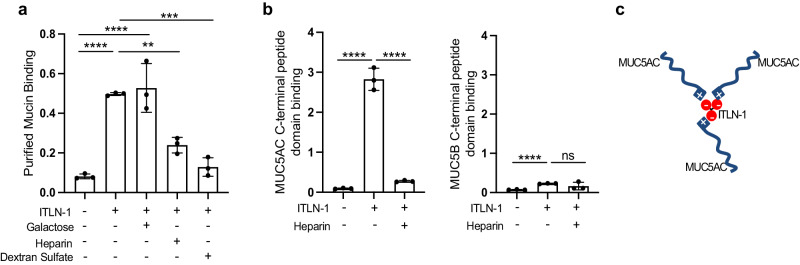
ITLN-1 binds to purified airway mucins and to the C-terminal domain of MUC5AC. **a** Bar graphs measuring binding of recombinant flag-tagged ITLN-1 protein, and the effect of co-incubation with galactose, heparin, or dextran sulfate, to purified human airway mucins (isolated and pooled from *n* = 5 participants). Data represent mean values ± SD; one way ANOVA with multiple comparisons **p = 0.0032, ***p = 0.0002, and ****p < 0.0001. **b** Binding assays measuring the ability of ITLN-1 protein, or co-incubated ITLN-1 + heparin, to bind to immobilized C-terminal peptides of MUC5AC (left) or MUC5B (right). Data representative of *n* = 3 independent experiments and bar plots represent mean values ± SD; Two-sided *t* test ****p < 0.0001, ns = not significant. **c** Illustration of the potential mechanism by which an ITLN-1 protein trimer could interact with the C-terminal domain of MUC5AC mucin molecules via electrostatic interactions. Source data are provided as a Source Data file.

Based on the MCM changes observed in the ITLN-1 KO, we hypothesized that ITLN-1 protein might bind to mucin monomers resulting in mucin cross-linking and thus altering the visco-elastic properties of mucus. To explore this, we evaluated the binding of recombinant human ITLN-1 protein to immobilized mucin proteins purified from the airway mucus of five donors. This assay indicated significant binding between ITLN-1 and the purified mucins (Fig. 3a). Considering that ITLN-1 has high affinity for galactofuranosyl residues, we next evaluated the ability of galactose to inhibit this binding. We found no significant inhibition of the ITLN-1:mucin interaction with the addition of galactose (Fig. 3a), confirming previous works showing that ITLN-1 has no affinity for mammalian glycans^11,12^. As ITLN-1 has a prominent negative charge, we considered whether charge-charge interactions could mediate this binding. Testing ITLN-1 binding to mucin protein in the presence of heparin or dextran sulfate, two compounds with high negative charge density, revealed that both reagents effectively decreased ITLN-1 binding to purified mucin proteins by 51.9% and 74.0%, respectively (Fig. 3a). Previously, heparin has been shown to bind the C-terminal tail of the intestinal mucin, MUC2^18,19^, and a similar domain is present in the C-terminal tail sequences of both the MUC5AC and MUC5B airway mucin proteins. Therefore, we first tested the ability of heparin to bind to human-purified mucins, and detected significant interaction between heparin and the human-purified airway mucins (SFig. 5a). We next tested binding of heparin and ITLN-1 to immobilized C-terminal peptides of MUC5AC and MUC5B, and found that individually, both ITLN-1 and heparin bound to C-terminal MUC5AC but not C-terminal MUC5B (Fig. 3b; SFig. 5b, c). Furthermore, the ITLN-1:C-terminal MUC5AC interaction was significantly inhibited by the addition of heparin, suggesting this interaction is electrostatic in nature (Fig. 3b). Together, these data support significant binding between ITLN-1 and airway MUC5AC, presenting the trimeric form of ITLN-1 as a potential crosslinker of mucins in pathologic airway mucus, and as a potential mechanism driving the ITLN-1 effects on mucociliary functions (Fig. 3c).

### *ITLN1* upregulation in T2-high asthmatics is strongly dependent on an eQTL variant

**Fig. 4:**
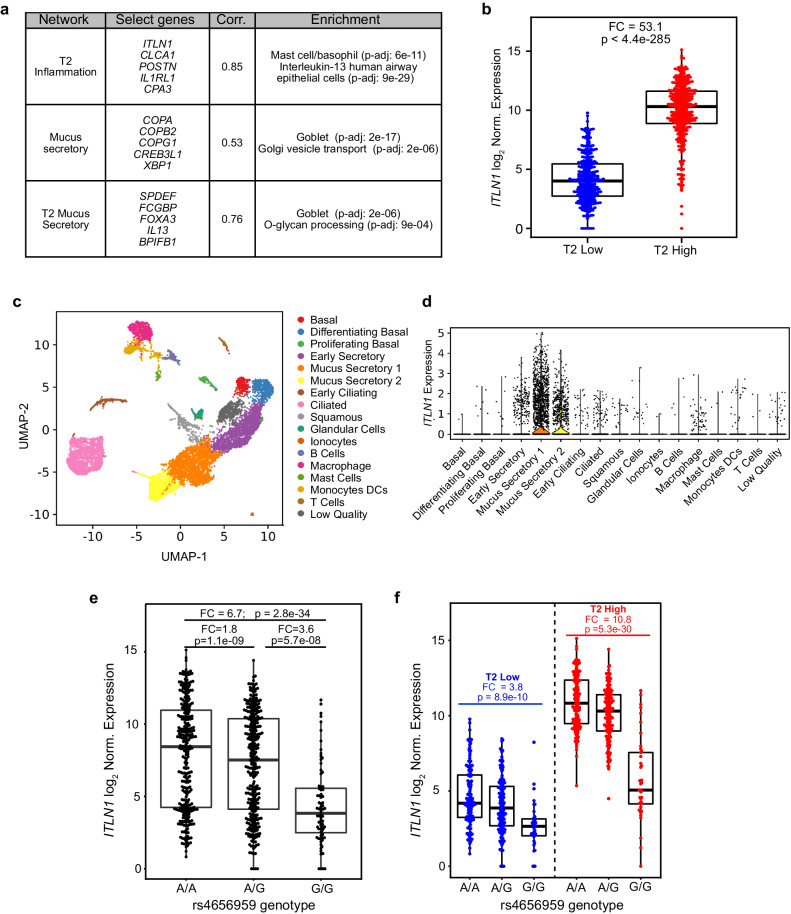
*ITLN1* is correlated with T2 inflammatory mucus secretory expression networks and is expressed by mucus secretory cells in vivo. **a** WGCNA conducted using gene expression from nasal airway epithelial brushings collected from 695 children in the GALA II asthma study found a strong correlation of *ITLN1* expression with T2 inflammation and mucus secretory networks. Select genes from each network, Pearson correlation between expression of *ITLN1* and network eigengenes, and top functional enrichments from each network are given. Enrichment *p* values were obtained from a one-sided Fisher exact test implemented in Enrichr. FDR-adjusted *p* values were obtained using the Benjamini–Hochberg method. **b** Box plots of log_2_-normalized *ITLN1* gene expression in GALA II nasal epithelial brushes stratified by T2 inflammation status (*n*: T2-low = 331, T2-high = 364). Fold change (FC) and two-sided *p* value from a Wald test was obtained from DESeq2. Box centers = median, upper and lower box bounds = 1st and 3rd quartiles, whiskers extend from these bounds up to 1.5 × IQR (inter-quartile range). All data points are overlain. **c** UMAP visualization of 11,515 cells from scRNA-seq of two dissociated bronchial airway epithelial brushings detailing the 17 cell types identified by SNN clustering through the Leiden algorithm. **d** Violin plots of log count per million (CPM)-normalized *ITLN1* expression across the 17 distinct cell types identified in bronchial airway brushings. **e** Box plots of log_2_-normalized *ITLN1* expression in GALA II stratified by the A/A (*n* = 292), A/G (*n* = 313), or G/G (*n* = 76) genotypes of the top eQTL variant rs4656959. Two-sided p-values were obtained from a Wald test in DESeq2. Box plots as defined in **b**. **f** Box plots of log_2_-normalized *ITLN1* expression in GALA II, stratified by T2 inflammation status and the genotypes of the top eQTL variant rs4656959 (T2-low *n*: A/A = 141, A/G = 148, G/G = 35 and T2-high *n*: A/A = 151, A/G = 165, G/G = 41). Two-sided *p* values were obtained from a Wald test in DESeq2. Box plots as defined in **b**. Source data are provided as a Source Data file.

We next sought to examine in vivo regulation of *ITLN1* airway expression. To do this, we analyzed published network gene expression data derived from RNA-seq data generated on the in vivo nasal airway epithelial brushings of 695 children in the Genes-Environments and Admixture in Latino Americans (GALA II) asthma study^20,21^. These children were assigned into one of two groups, T2-high (*n* = 364) and T2-low (n = 331), by hierarchically clustering (*K* = 2) participants based on their expression of the identified T2 inflammatory network^20,21^. This T2 inflammatory network contained the canonical T2 epithelial biomarker genes, *POSTN*, *CLCA1*, *CPA3*, *CCL26*, *DPP4*, and *IL1RL1*. Importantly, we found that the *ITLN1* gene was in this T2 inflammation network, and that its expression was highly correlated with overall T2 network expression (*r* = 0.85, *p* = 1.5e-191; Fig. 4a). In fact, *ITLN1* expression was 53.1-fold higher among T2-high vs. T2-low patients (*p* < 4.4e-285; Fig. 4b). Additionally, we found *ITLN1* expression was highly correlated with that of two mucus secretory networks (Fig. 4a). We also observed a strong positive correlation between *ITLN1* and the T2-associated mucin, *MUC5AC* (*r* = 0.42, *p* = 1.8e-30; SFig. 6a), and a moderate negative correlation between *ITLN1* and *MUC5B* expression (*r* = −0.22; *p* = 9.1e-09; SFig. 6b). These data are consistent with our in vitro data showing *ITLN1* is a mucus secretory cell gene, more associated with MUC5AC expressing cells.

To confirm the airway epithelial cell types expressing *ITLN1* in vivo, we performed a single-cell RNA-seq (scRNA-seq) analysis of bronchial airway epithelial brushings from two children with asthma. Performing shared nearest-neighbor clustering of the 11,515 cells, we identified 17 distinct cell clusters and then assigned cell types to these clusters by overlaying known epithelial cell type marker genes with differentially expressed genes for each cell cluster (Fig. 4c; Supplementary Data 2). This analysis identified two mucus secretory cell populations, both of which highly expressed *ITLN1*, compared to all other cell populations (Fig. 4d). These results confirm and extend to the in vivo bronchial epithelium that *ITLN1* is highly expressed by mucus secretory cells.

We next evaluated the role of population genetic variation in regulating airway *ITLN1* expression through examination of *ITLN1* eQTL data extracted from the results of a published genome-wide nasal airway cis-eQTL analysis performed on the same children in the GALA II study^21^. This analysis identified 523 genetic variants that were significantly associated with *ITLN1* expression, which were reduced by stepwise forward-backward regression analysis to two variants with independent effects on *ITLN1* expression. The variant with the most significant effect on expression (rs4656959, *p* = 2.0e-47, MAF 0.34), is located 4 kb upstream of the *ITLN1* gene, and marked a linkage disequilibrium block of 79 variants (with *r*^2^ > 0.7). This *ITLN1* rs4656959 variant resulted in a dramatic 6.7-fold decrease in expression between the AA and GG genotypes (*p* = 2.8e-34; Fig. 4e). We performed multivariable modeling of *ITLN1* expression as a function of T2 inflammation status and rs4656959 genotype to investigate the independent and interactive effects of these factors. This analysis confirmed strong effects of both the rs4656959 variant (*p* = 9.5e-08) and T2 status (*p* = 1.99e-91; Supplementary Data 3) on *ITLN1* expression. Additionally, we found the magnitude of the genotype effect was dependent on T2 inflammation (interaction *p* = 3.1e-04), as the *ITLN1* expression between AA versus GG genotype participants was 3.8-fold lower among T2-low patients (*p* = 8.9e-10), but was 10.8-fold lower among T2-high patients (*p* = 5.3e-30; Fig. 4f). These results reveal the strong role that both T2 inflammation and the rs4656959 variant play in the regulation of *ITLN1* gene expression in vivo. Moreover, we found the *ITLN1* eQTL variant was not associated with either *MUC5AC, MUC5B*, or T2 mucus secretory network expression, indicating that this loss of *ITLN1* expression did not inhibit T2 inflammation-directed mucus metaplasia (SFig. 6c–e).

### The rs4656959 variant is associated with loss of ITLN-1 protein expression and airway epithelial secretion

**Fig. 5:**
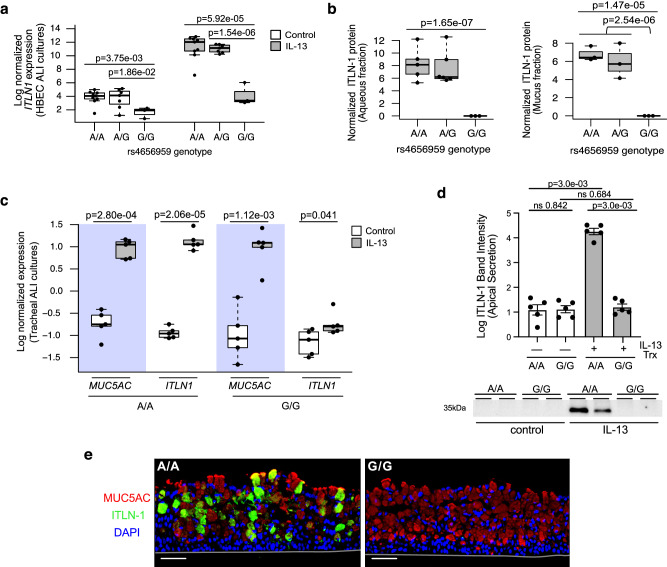
*ITLN1* rs4656959 variant eliminates ITLN-1 protein expression in airway epithelia. **a** Normalized *ITLN1* gene expression from paired HBEC ALI cultures (*n* = 19 donors) stratified by treatment (control vs. IL-13) and by *ITLN1* rs4656959 genotype for both treatments (*n*: A/A = 9, A/G = 7, and G/G = 3). Two-sided *p* values for indicated differences are based on an exact test (edgeR). Box centers = median, upper and lower box bounds = 1st and 3rd quartiles, whiskers extend from these bounds up to 1.5 × IQR (inter-quartile range). All data points are overlain. **b** Box plots showing normalized ITLN-1 protein secretion measured in aqueous (left; *n*: A/A = 5, A/G = 6, and G/G = 3) and mucus (right; *n*: A/A = 3, A/G = 3, and G/G = 3) fractions from apical washes of IL-13-stimulated HBEC ALI cultures. Two-sided *p* values are based on an exact test (edgeR). Box plots as defined in **a**. **c** Normalized *ITLN1* and *MUC5AC* expression measured by qPCR from tracheal mucociliary ALI cultures stratified by treatment (control-white or IL-13-gray) and by *ITLN1* rs4656959 genotype (*n* = 5 donors/genotype). Two-sided p-values are based on linear mixed model *t* tests with donor as random intercept using Satterthwaite approximation of degrees of freedom. Box plots as defined in **a**. **d** Western blot analysis measuring ITLN-1 in apical washes from control or IL-13-stimulated tracheal ALI cultures with the A/A or G/G genotype of rs4656959 (*n* = 5 donors/genotype). Western blot image is representative of data from all donors. Band intensity data reflect all conditions from all donors (*n* = 5 donors/genotype). Data represent mean value ± SEM; Two-sided *p* values calculated using Student’s *t* test. **e** Immunofluorescent labeling of IL-13-treated tracheal ALI cultures for ITLN-1 (green), MUC5AC (red), and nuclei (DAPI); images are representative of all 10 donors analyzed; scale bar 50 μm. Source data are provided as a Source Data file.

We next genotyped and evaluated the effect of the rs4656959 variant on *ITLN1* expression among our HBEC ALI cultures characterized in Fig. 1 (*n* = 19), of which 9 were the AA, 7 were the AG, and 3 were the GG genotype. We found a strong genotypic effect, with 6.9-fold lower *ITLN1* expression in rs4656959 GG relative to AA genotype cultures under baseline conditions (*p* = 3.8e-03), and 131-fold lower *ITLN1* expression between genotypes when treated with IL-13 (*p* = 5.9e-05; Fig. 5a). Furthermore, we found that ITLN-1 protein secretion was induced by IL-13 among both AA and AG genotype cultures, while absent within the aqueous fraction collected from GG genotype cultures, with a similar result observed for the mucus fraction (Fig. 5b).

To confirm these findings, we identified five patient samples, each with AA and GG genotypes for the rs4656959 variant from our bank of tracheal airway epithelial basal cells, which we validated to exhibit IL-13 responses similar to bronchial cells (SFig. 7). These cells were differentiated into mucociliary cultures at ALI under either control or IL-13 treatment conditions, and targeted gene expression was measured by qPCR analysis. We found poor expression of *ITLN1* in AA control cultures, which was increased 117.1-fold with IL-13, while *ITLN1* expression was very minimally increased by IL-13 treatment among GG cultures (Fig. 5c). In contrast, *MUC5AC*, a key marker of IL-13-induced mucus metaplasia, was robustly induced among both the AA (FC = 49.8, *p* = 2.8e-04) and GG (FC = 89.7, *p* = 1.1e-03) genotype groups (Fig. 5c). Western blot analysis on apical washes from these tracheal cultures revealed that all five of the AA donors significantly upregulated apical ITLN-1 protein secretion following IL-13 stimulation (*p* = 3.0e-03; Fig. 5d), while secreted ITLN-1 protein was absent in all control and IL-13-stimulated cultures with the GG genotype. This total absence of ITLN-1 protein among GG donors was further confirmed by immunofluorescent labeling of histological sections from these cultures (Fig. 5e). Taken together, these results demonstrate that the rs4656959 GG genotype drives the elimination of ITLN-1 protein, whether cellularly localized or apically secreted, even while a robust IL-13-induced mucus metaplasia is maintained.

### The *ITLN1* rs4656959 variant is protective against T2 inflammation-driven airway mucus plugging in severe asthmatics

Previously, we found that 58% of severe asthmatics in the Severe Asthma Research Program (SARP) cohort exhibited mucus plugging of lung segments^8^. Moreover, high mucus plug scores (>4 plugged lung segments) were associated with high sputum eosinophil levels, a clinical marker of T2 airway inflammation^8^. Based on our finding that ITLN-1 protein regulates mucociliary movement in an in vitro model of T2 inflammation-induced mucostasis, we investigated whether the rs4656959 *ITLN1* eQTL might modulate risk of mucus plugging among SARP participants.

**Fig. 6:**
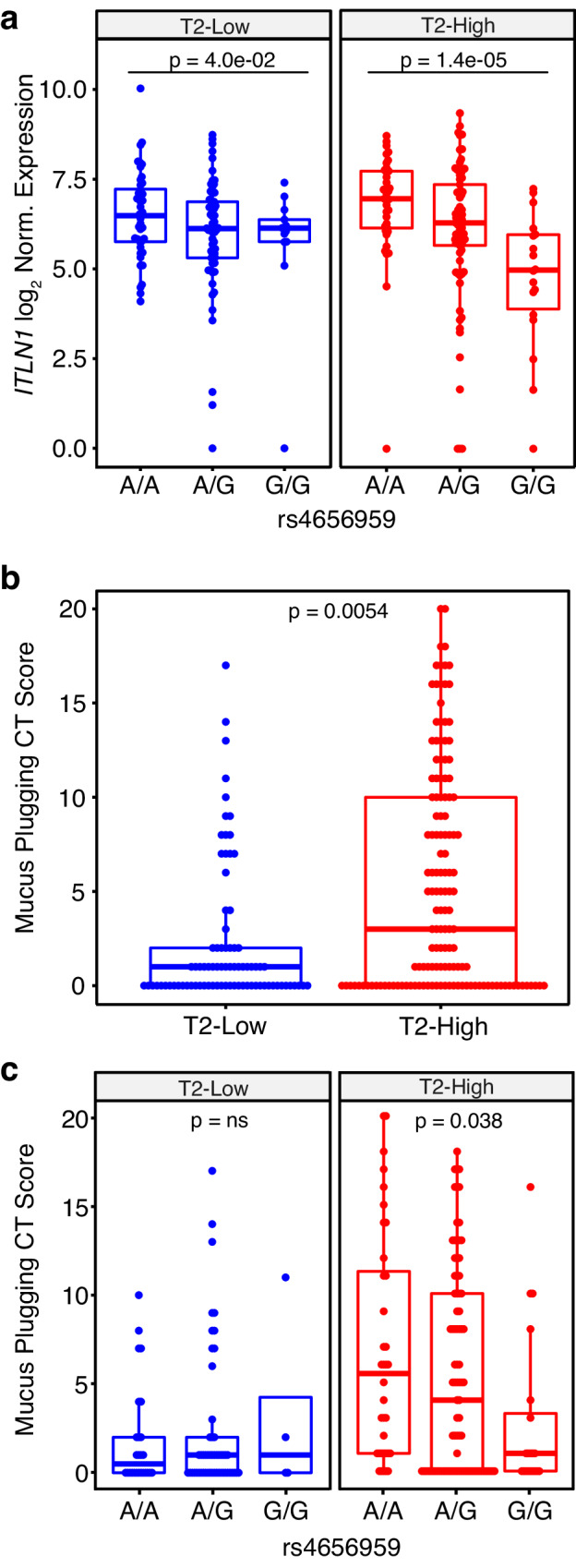
*ITLN1* rs4656959 is associated with lower mucus plug scores in T2-high asthmatics. **a** Box plots of log_2_-normalized *ITLN1* expression from 249 participants in the SARP asthma cohort stratified by T2 status and *ITLN1* rs4656959 genotype; *n*: T2-low participants n: A/A = 43, A/G = 61, G/G = 13 and T2-high participants *n*: A/A = 37, A/G = 77, G/G = 18. Two-sided *p* values for indicated differences were obtained from a Wald test in DESeq2. Box centers = median, upper and lower box bounds = 1st and 3rd quartiles, whiskers extend from these bounds up to 1.5 × IQR (inter-quartile range). All data points are overlain. **b** Box plots of mucus plug scores (2 measurements per participant) from SARP, stratified into T2-low (*n* = 41 participants) and T2-high (*n* = 71 participants) groups based on sputum RNA-seq expression profiles. Two-sided *p* values were obtained by fitting negative binomial mixed model implemented in SAS PROC GLIMMIX (METHOD = RSPL; DDFM = KENWARDROGER2) with subjectID as random effect. Box plots as defined in **a**. **c** Box plots of mucus plug scores (2 measurements per participant) from SARP, stratified by both T2 status and *ITLN1* rs4656959 variant genotype; T2-low participants n: A/A = 13, A/G = 26, G/G = 2 and T2-high participants *n*: A/A = 18, A/G = 43, G/G = 10. Two-sided *p* values were obtained by fitting a negative binomial mixed model implemented in SAS PROC GLIMMIX (METHOD = RSPL; DDFM = KENWARDROGER2) with subjectID as random effect. Box plots as defined in **a**. Source data are provided as a Source Data file.

We first sought to verify the role of both T2 inflammation and the rs4656959 variant on *ITLN1* expression in lung airway samples by utilizing RNA-seq data generated on sputum cell pellets from 249 severe asthmatics within the SARP cohort. Participants were stratified into T2-high (*n* = 132) and T2-low (*n* = 117) groups by hierarchical clustering using expression of genes in the T2 inflammation network (Supplementary Data 4, see Methods). The rs4656959 variant was significantly associated with sputum *ITLN1* expression among both T2-low (FC = 1.8, *p* = 4.0e-02) and T2-high patients (FC = 3.2, *p* = 1.4e-05; Fig. 6a), although this association was weaker, likely due the low and highly variable epithelial contribution to sputum samples, which confounds this analysis. Similar to our analyses in nasal brushings in the GALA II study, we found no association between either *MUC5AC* or *MUC5B* expression and the *ITLN1* eQTL (SFig. 8a, b). We observed a moderate positive correlation between *ITLN1* and *MUC5AC* expression (*r* = 0.24; *p* = 9.7e-05; SFig. 8c), whereas *ITLN1* was not correlated with *MUC5B* expression (*r* = 0.11; *p* = 0.067; SFig. 8d), reinforcing the co-expression of *ITLN1* and *MUC5AC*.

CT-based mucus plug scores were available at two timepoints (3 years apart) on 112 SARP asthmatics. 37% of T2-low patients exhibited a score above 0 versus 61.5% of T2-high patients, and the mean plug score was 2.05 for T2-low patients versus 5.22 for T2-high patients (*p* = 0.0054; Fig. 6b). With the low frequency of mucus plugs among T2-low participants we found no relationship between plug score and rs4656959 genotype. However, among T2-high participants we found a significant additive relationship between the rs4656959 genotype and mucus plugging (*p* = 0.038), with the GG genotype patients, associated with lower *ITLN1* expression, exhibiting lower average mucus plug scores (AA = 6.5, AG = 5.3 GG = 2.7; Fig. 6c).

## Discussion

The pathobiology underlying mucus obstruction of the airways and susceptibility to this phenotype remain some of the most enigmatic aspects of asthma and other obstructive lung diseases^22^. Most studies in this area have focused on the amount of mucus secreted (mucus hypersecretion), as well as distortions in both the concentration and ratio of MUC5AC and MUC5B mucin proteins in airway mucus^10,23^. However, our recent finding that IL-13 drives the formation of a distinct mucus secretory cell type, with a unique secretome, suggests that non-mucin proteins might contribute to mucus obstruction experienced by T2-high asthmatics^5^. Here, we characterize *ITLN1*, finding it is induced in a switch-like fashion in the airway epithelium by IL-13 as part of a metaplastic mucus secretory network and that ITLN-1 protein is incorporated into the airway mucus gel. Moreover, we find that ITLN-1 can directly bind MUC5AC through electrostatic interactions and that its deletion partially reverses IL-13-induced mucostasis. Importantly, we report a common eQTL variant for the *ITLN1* gene. This variant drives a dramatic reduction in both gene and protein expression from the airway epithelium, which is associated with protection from mucus plugging, specifically among T2-high asthmatics. Our findings document one of the first non-mucin-secreted airway proteins to be implicated in airway mucus obstruction in asthma.

In contrast to the intestinal epithelium, where *ITLN1* is constitutively expressed by mucus secretory cells^12,24^, we find little *ITLN1* expression in the airway epithelium in the absence of T2 inflammation. In the airway, we found that *ITLN1* is expressed specifically within a gene network activated by a distinct population of IL-13-induced mucus secretory cells, explaining its switch-like induction in the airway of T2-inflamed individuals, particularly evident in our in vitro protein assays. Our observation that airway epithelial *ITLN1* expression is regulated by IL-13 confirms reports from several mouse and human studies^25–27^. But the degree to which this IL-13 regulation occurs is striking - *ITLN1* expression was induced by IL-13 more than 100-fold in primary bronchial and tracheal airway epithelial cells in culture and by 53-fold in brushed nasal airway epithelia from children with T2-high inflammation. Notably, 52% of variation in *ITLN1* expression was explained by T2 status in children.

Deletion of ITLN-1 protein partially reverses the loss of mucociliary motion and ciliary beat frequency characteristic of IL-13-activated airway epithelial cells. This result led us to consider whether ITLN-1 might directly bind mucin proteins to alter the biophysical properties of mucus. Similar to prior experiments^13^, we found that ITLN-1 did not bind human airway mucins in a galactose-dependent manner, consistent with prior findings that ITLN-1 selectively binds microbial glycan epitopes^11,12^. Instead, we discovered that the negatively charged ITLN-1 binds the positively charged C-terminus of MUC5AC via electrostatic interactions. ITLN-1 did not bind the C-terminus of MUC5B even though, like MUC5AC, MUC5B has positively charged amino acids in its C-terminal domain (CTCK)^18^. Given the homotrimeric nature of ITLN-1, it is possible that ITLN-1 could crosslink MUC5AC proteins via electrostatic interactions (Fig. 3c); however, further validation including stoichiometric studies are needed to validate this illustration. Nevertheless, disulfide mucin cross-links increase the elastic behavior of mucus^28^, and it is plausible that ITLN-1 could also cross-link mucin polymers to stiffen the airway mucus gel.

We report a naturally occurring correlate of our experimental ITLN-1 KO in the form of a common human genetic variant that greatly diminishes *ITLN1* airway gene expression. Specifically, we find that the induction of ITLN-1 by T2 inflammation is largely dependent on the rs4656959 genetic variant, constituting a striking gene-environment interaction that controls airway abundance of ITLN-1. Furthermore, in independent sets of primary bronchial and tracheal epithelial cultures, ITLN-1 protein is not produced or secreted by rs4656959 GG donor cultures, even in the presence of IL-13, suggesting that this variant functions as a protein knockout. Interestingly, the rs4656959 SNP is part of an 84-SNP linkage disequilibrium block (*r*^2^ > 0.8) in Europeans, which stretches up- and downstream of *ITLN1* and has been associated with Crohn’s disease in GWAS studies^15,16^. In stark contrast to our findings in the airway epithelium, a study investigating the effects of this Crohn’s disease risk haplotype on *ITLN1* expression in the ileum and colon found that *ITLN1* did not function as an eQTL in intestinal tissue^24^. This European haplotype also contains an ITLN-1 valine-to-aspartic acid coding variant (rs2274907), hypothesized to functionally mediate the Crohn’s disease association. However, recent investigation of ITLN-1 protein isoforms derived from this variant, found no isoform-associated alterations to glycan binding kinetics, oligomerization, or protein stability^24^. Additionally, we find only 19 variants are in strong LD with rs4656959 in our Puerto Rican population, of which rs2274907 is not one. Further experiments will be needed to confirm that rs4656959, and not other linked SNPs, is responsible for the observed expression effects and to determine the molecular mechanism by which the causative variant alters expression.

Occlusion of airways by mucus plugs has long been recognized as a cause of airway obstruction in acute severe asthma (including fatal cases of asthma). More recently, we have quantified airway mucus plugging using a bronchopulmonary segment-derived mucus plug score and found that high airway mucus plug scores are common in chronic severe asthma and are strongly associated with measures of type 2 inflammation and airflow obstruction in both cross-sectional and longitudinal studies^8,29^. Notably, using airway mucus plug scores in asthma patients genotyped for the *ITLN1* eQTL variant, rs4656959, we discovered that rs4656959 is associated with protection from mucus plugging, specifically among T2-high patients. This finding suggests that ITLN-1 mediates pathologic mucus plug formation in T2-high asthma.

We acknowledge the limitations of this study, including its uncertainty regarding a mechanism by which ITLN-1 contributes to mucositis observed during in vitro and in vivo mucus plugging. Moreover, given the novelty of measuring the mucus-plugging phenotype, our genetic association with mucus-plugging was performed in a small number of participants and thus will need to be replicated. However, the T2 specificity and direction of our genetic association result are consistent with our other findings, including those related to IL-13-induced *ITLN1* upregulation, genetic direction of effect, in vitro knockout phenotype, and mucin binding, giving us confidence this is a valid finding.

In conclusion, our results suggest that ITLN-1 contributes to T2 inflammation-induced airway mucostasis and airway plugging and that a common *ITLN1* eQTL variant mediates the risk of poor mucus-related outcomes in patients with severe T2-high asthma.

## Methods

### Human participant information

All research conducted in these studies complies with all relevant ethical regulations. Human bronchial and tracheal airway epithelial cells from adult participants were banked in a de-identified fashion and obtained for research purposes from the National Jewish Health (NJH) Live Cell Core. The NJH Live Cell Core is an institutional review board-approved study (HS-2240) for the collection of tissues from consented patients for researchers at NJH. For tracheal epithelial cell isolation, human lung specimens were obtained from de-identified lung donors whose lungs were not suitable for transplantation from the International Institute for the Advancement of Medicine (Edison, NJ), and Donor Alliance of Colorado. Written consent for research on tracheal and lung specimens was obtained by the Organ Procurement Organization prior to organ donation. The National Jewish Health Institutional Review Board (IRB) approved the research on lung cells under IRB protocol HS-3209. Nasal airway epithelial brushes were collected from participants recruited as part of the Genes-environments and Admixture in Latino Americans II (GALA II) childhood asthma study to be used in genome-wide genetic and genomic analysis, which was approved by local institutional review boards (UCSF, IRB number 10–00889, Reference number 153543, NJH HS-2627). All participants and their parents provided written informed assent and written informed consent for sample collection and use of the samples in genetic and genomic analyses^30,31^. Bronchial airway brushings used for scRNA-seq were collected following informed consent and assent as part of the Epithelial Barriers in Asthma, Eczema, Food Allergy, and GERD study approved by the BRANY IRB for National Jewish Health under protocol HS-3255-528. Airway tracheal epithelial cells used to model the IL-13 response compared to bronchial responses were harvested from the tracheas of organ donors obtained from the California Transplant Donor Network^32^. Sputum specimens and mucus plug scores were collected following informed consent from participants, into the Severe Asthma Research Program (SARP3) study, and the study was approved by the IRBs at all participating centers: Brigham and Women’s Hospital (Partners Human Research Committee, 2012P001528), Boston Children’s Hospital (Boston Children’s Hospital IRB, IRB-P00004759), University of Wisconsin (University of Wisconsin Madison Health Sciences IRB, 2012-0571-CP002), University of Pittsburgh (University of Pittsburgh IRB, PRO12070359), Washington University (Washington University in St. Louis IRB, 201206102), University of California—San Francisco (University of California—San Francisco IRB, 12-09392; 12-09556), University of Virginia (IRB for Health Sciences, 16400), Cleveland Clinic (Cleveland Clinic IRB, 6185), Virginia Commonwealth University (Virginia Commonwealth University IRB, HM14883), Rainbow Babies (University Hospital IRB, 09-12-08), Wake Forest (Wake Forest IRB, IRB00021507), Emory University (Emory University IRB, IRB00058103). Summary of variables in each cohort including age, sex, health status, and T2 status and *ITLN1* rs4656959 genotype (where applicable) are reported in Supplementary Data 5.

### ALI culture and IL-13 stimulation of primary bronchial and tracheal airway epithelial cells

For both primary human bronchial (*n* = 19) and tracheal (*n* = 5 per *ITLN1* genotype group) epithelial samples, basal cells were cultured for expansion on irradiated NIH 3T3 mouse fibroblasts (ATCC, CRL-1658) using a modified Schlegel method as previously described^33,34^. Primary basal bronchial cells were seeded onto 6.5 mm, 0.4 μm pore transmembrane inserts (Corning; #3470) pre-coated with PureCol bovine collagen (2 × 10^4^ cells/insert) in ALI expansion medium supplemented with Y-27632. Primary tracheal cells were seeded onto 6.5 mm 24-well polyester Transwell inserts with 0.4 μm pore size (4 × 10^4^ cells/insert) in PneumaCult Expansion Plus medium (StemCell Technologies) supplemented with Y-27632. Cultures were air-lifted upon the development of an intact monolayer, and basolateral media was changed to PneumaCult ALI (PC-ALI) media to allow for epithelial differentiation. At day 11 post-airlift, paired culture inserts in triplicate (*n* = 3 per treatment condition) were stimulated with either IL-13 (10 ng/ml) or BSA-supplemented media (mock control) daily for 10 days and harvested for experiments as described below. For tracheal ALI stimulations performed in SFig 7, tracheal basal cells (*n* = 40) were harvested, and cells were expanded for 1 passage in Basal Epithelial Growth Media (Lonza) and then plated to 12 mm diameter transwells^35^. Cells were grown for 21–23 days in 2% Ultroser G (Pall Corporation) in DMEM/F12 media and then stimulated with media alone or IL-13 (10 ng/ml; R&D Systems) every 24 hours for 2 days. Cultures were harvested, and RNA was isolated for gene expression analysis.

### Immunofluorescence labeling and confocal imaging

Following mock or IL-13 stimulation, ALI cultures were fixed in 3.2% paraformaldehyde/PBS prior to labeling. For histological section labeling, fixed ALI inserts from eight paired donor cultures were paraffin-embedded and sectioned. Histology sections were stripped of paraffin using HistoChoice Clearing Agent (Sigma), rehydrated using a decreasing gradient of alcohol washes (100%, 90% 70%, 50%, 30%, 0%), and antigen retrieval conducted using Citric Acid-based Antigen Unmasking Solution pH 6.0 (Vector Laboratories). Prior to labeling, all samples were blocked and permeabilized for 30 minutes using 3% BSA/0.1% Triton X-100 in tris-buffered saline (TBS). Primary labeling was conducted using 3% BSA/0.1% Triton X-100 in TBS for 1 hour with antibodies against MUC5AC (1:500; Fisher Scientific MA1-21907), MUC5B (1:200; SantaCruz sc-20119), or ITLN-1 (1:200; AbCam ab118232). Samples were washed twice using TBS/0.1% Triton X-100 (TBST), and secondary labeling was conducted using DAPI (1:1000), AlexaFluor488 goat anti-rabbit IgG (1:500; Thermo Scientific, A-11008) and AlexaFluor594 goat anti-mouse IgG (1:500; Thermo Scientific, A-11005) for 30 minutes. Slides were washed twice in TBST and mounted with Vectashield HardSet Antifade Mounting Media (Vector Laboratories). Fluorescent images of histological sections were acquired using a Revolve R4 microscope (Echo Labs; EchoPro app v6.4.1). Whole mount ALI sections from three donors were fixed, blocked, and labeled as described above, and Z-stack images were captured using a Zeiss LSM700 Confocal microscope. Confocal image analysis and annotation were performed using Zeiss ZEN Blue Lite analysis software.

### Proteomic sample preparation and mass spectrometry

For aqueous fraction proteomic analyses, apical washes from paired BSA (mock) and IL-13-treated ALI cultures were collected from inserts cultured in triplicate from a subset of donors from the HBEC experiments (*n* = 14) individually in Fisher 2 ml low binding microfuge tubes, and samples were centrifuged at 225 × *g* at 4 °C to remove intact cells from the washes. Supernatants were collected from each sample tube, and the visible insoluble (i.e., mucus) fraction was removed from the soluble (aqueous) fraction. The soluble apical fraction was mixed with 3× volume of ice-cold LC/MS grade methanol (Fisher Scientific; A456-500) followed by incubation at −80 °C for 1 hour. Proteins were then pelleted by centrifugation at 18,000 × *g,* and the supernatant was removed. Protein pellets were dried in a SpeedVac at 45 °C and frozen at −80 °C. For trypsin digestion, protein pellets from 2 of the harvested culture inserts per condition were processed and pooled for analysis as described below. Samples were denatured and reduced using 50% trifluoroethanol in 50 mM ammonium bicarbonate with 0.1 mM DTT at 65 °C for 45 minutes. Proteins were then alkylated for 1 hour in the dark using 0.32 mM iodoacetamide (IAA), and excess IAA was then reduced using 0.08 mM DTT for 1 hour in the dark. Samples were diluted with 400 μl of 25 mM ammonium bicarbonate buffer and digested with 0.3 μg of trypsin for 5 hours at 37 °C. Digests were then stopped using 2 μl of formic acid and duplicate samples were combined and dried at 45 °C in a SpeedVac concentrator, and dried samples were stored at −70 °C. For mass spectrometry, the soluble fraction samples were first resuspended with 10 μl of 3% Acetonitrile + 0.1% formic acid, and the total sample was analyzed using a nanoAdvanced UPLC (Bruker) with a 15 cm × 100 μm ProntoSil C18AQ column and 2 cm trap column (nanoLCMS Solutions). Mobile phase was H_2_O + 0.1% FA (A) and acetonitrile + 0.1% FA (B), peptides were separated using a gradient of 2–40% B over 30 minutes at a flow rate of 800 nL/minute and with the column temperature kept constant at 40 °C. The column was connected through a Captive Spray nano source to an Impact Q-TOF (Bruker). Data were collected over a mass range of 150–2200 m/z using a 1 Hz MS scan and a total duty cycle time of 2 seconds. Data were processed using DataAnalysis 4.2 (Bruker), compounds were searched against the SwissProt database using Mascot 2.4 (Matrix Science) with the percolator algorithm, and protein and peptide results were assessed and filtered with ProteinScape 3.0 (Bruker). Raw peptide counts were normalized to the total number of peptides measured within each sample, and differential presence of ITLN-1 was analyzed by EdgeR analysis using *p*-value < 0.05 as a significance cutoff.

For mucus fraction proteomics, apical washes from paired BSA (mock) and IL-13-treated ALI cultures were collected from inserts cultured in triplicate from a subset of donors used in the HBEC experiments (*n* = 9) individually in Fisher 2 ml low binding microfuge tubes and samples were centrifuged at 225 × *g* at 4 °C to remove intact cells from the washes. Supernatants were collected from each sample tube and the visible insoluble (i.e., mucus) fraction was recovered, dried in a SpeedVac at 45 °C, and stored at −80 °C. Frozen mucus fraction pellets from 2 of the harvested culture inserts per condition were processed as detailed below and pooled for analysis. Samples were processed and digested following the Preomics Sample Preparation Kit for Pelleted Cells and Precipitated Proteins v2.5. Briefly, mucus pellets were suspended in 25 μl of Lyse buffer, vortexed for 5 min, then incubated at 95 °C for 10 min, and vortex mixed prior to pooling and sonication for 10 cycles of 30 sec On/Off. Digest buffer was diluted 1:4 with Resuspend buffer, and then mixed 1:1 with sample in Lyse buffer prior to digestion for 3 hrs and 10 min at 37 °C. Sample digestion was stopped, washed twice, and eluted. Final eluates were dried in a speedvac at 45 °C and stored overnight at −20 °C. Samples were suspended in Preomics Load buffer and bath sonicated for 10 cycles of 30 sec On/Off and stored at −70 °C. Samples were analyzed using LCMS methods similar to a previous publication^36^. Samples were loaded onto a 2 cm PepMap 100, nanoviper trapping column and chromatographically resolved online using a 0.075 × 150 mm, 3.0 µm EASY-Spray PepMap RSLC C18 reverse phase nano column (Thermo Scientific) using an Ultimate 3000 RSCLnano LC system (Thermo Scientific). Mobile phases consisted of water + 0.1% formic acid (A) and 100% acetonitrile + 0.1% formic acid (B). Samples were loaded onto the trapping column at 5.0 μL/min for 4 minutes at initial condition before being chromatographically separated at a flow rate of 300 nl/min using a gradient of 3–7% B over 3 minutes, 7-22% B over 37 minutes, and 22-33% B over 5 minutes for a total 45 minute gradient at 40 °C. The gradient method was followed by a column wash at 70% B for 5 minutes. Data were collected on an Orbitrap Eclipse (Thermo Scientific) operated using intensity-dependent CID MS/MS to generate peptide ID’s. MS1 spectra were acquired in the Orbitrap (Resolution = 120k; AGQ target = 100%; MaxIT = Auto; RF Lens = 30%; mass range = 350–1500; Profile data). Precursors selected for MS/MS were filtered by MIPS model set to peptide with an intensity threshold of 15000 and only charge states 2–6 were allowed. Dynamic exclusion was employed for 14 s excluding all charge states for a given precursor. MS2 spectra were collected using CID in the linear ion trap (Isolation window = 1 m/z [quadrupole], rate = rapid; AGQ target = Standard; MaxIT = Dynamic; CID = 35%). Data were searched and extracted using SEQUEST HT and the label-free quantitation workflow in Proteome Discover software version 2.5.0.400 utilizing the minora feature detector, feature mapper and precursor ions quantifier algorithms. Spectra were searched against the SwissProt *Homo sapiens* database allowing up to 2 missed tryptic cleavages with fixed carbamidomethyl (C) and dynamic deamidated (N,Q) and oxidation (M) modifications. The monoisotopic peptide mass tolerance allowed was ± 6.0 ppm and the MS/MS tolerance was ±0.4 da. Peptides were adjusted to a 1% false discovery rate (FDR) using the percolator algorithm. Additionally, only high-confidence peptides were allowed for protein scoring and a protein FDR cutoff of 1% was also used. A signal to noise (S/N) threshold of 3 was set for minora feature detector and peptide spectrum match (PSM) confidence levels were set to high for feature ID linking. A coarse retention time (RT) alignment of data was performed using max RT shift of 10 min and mass error of 6 ppm. Following RT alignment, a RT tolerance of 1.4 min, mass tolerance of 6 ppm, and minimum S/N threshold of 5 were set for feature linking and mapping. Precursor quantification was based on ion intensity. Raw peptide level abundance and identification data were exported from proteome discover and filtered to peptides found in 100% of samples in 1 of 6 conditions. Peptide-level data were then rolled up into protein level data by summing the abundance of all unique peptides for a specific protein. Raw peptide counts were normalized to the total number of peptides measured within each sample, and differential presence of ITLN-1 was analyzed by EdgeR analysis using *p*-value < 0.05 as a significance cutoff.

### Cell type deconvolution

To estimate cell type proportions within the HBEC ALI RNA-seq data, we used CIBERSORTx^37^. We first created a signature matrix, which defines the genes whose expression characterizes cell types of interest, using scRNA-seq expression data consisting of 8381 cells and representing six major bronchial epithelial cell populations (basal, differentiating basal, early secretory, mucus secretory 1, mucus secretory 2, and ciliated cells). These data were based on bronchial epithelial brushings from two pediatric asthma patients (same data shown in Fig. 4c). Signature matrix generation used a minimum expression threshold of 0.5 and otherwise, default parameters. We then deconvolved the bulk HBEC ALI RNA-seq dataset based on the signature matrix to obtain cell type proportions, correcting for batch differences using S-mode.

### Lentiviral CRISPR-Cas9 gene editing of tracheal airway epithelial cells

The design of the CRISPR targeting guide sequences, addition of adapters and cloning into the lentiCRISPR plasmid backbone, propagation and titration of lentivirus, and AEC transduction and selection were performed as previously described^38,39^. The gRNA sequences were designed using the CRISPRdesign (version 1) tool offered by the Zhang lab at MIT (crispr.mit.edu) and chosen to target exon 2 of the *ITLN1* gene (IDT; Forward guide: ITLN1-75ex2gRNAFwd 5′-CACCGTTTCTCATAGCGACCACCAG-3′; Reverse guide: ITLN1-75ex2gRNARev 5′-AAACCTGGTGGTCGCTATGAGAAAC-3′; Fig. 2a). A scrambled sequence was designed and used as a control in parallel for all CRISPR gene editing experiments (forward scramble guide 5′- CACCGCGTGCTCCGTTCGCGCTTC-3′; reverse scramble guide 5′-AAACGAAGCGCGAACGGAGCACGC-3′). Screening primers were designed over the CRISPR-Cas9-targeted region of the *ITLN1* gene for high-resolution melt curve analysis using MeltDoctor HRM Master Mix (ThermoFisher) on a QuantStudio 6 Flex Real-Time PCR System (Life Technologies). Inference of CRISPR Edits (ICE) analysis was performed using the ICE Analysis tool v3.0 provided by Synthego^40^. DNA was isolated from control scrb- and ITLN1-targeted expanded basal cells prior to being seeded to transwell inserts. PCR amplification was performed using 25 ng of input DNA from each sample, with 0.2 μM of ITLN1_ICE1_Fwd primer (5′-GGCTGGAAGGTGACACAGTT-3′) and 0.2 μM ITLN1_ICE1_Rev (5′-CCCAAAACCAACACCAACTC-3′) primers using CloneAmp HiFi PCR PreMix (Takara Bio) as per manufacturer’s instructions. PCR amplicons were purified using the QIAquick PCR Purification Kit (Qiagen), and submitted for Sanger sequencing using the ITLN1_ICE1_SeqF primer (5′-GACACAGTTCTTGCCACAGC-3′). Early passage primary tracheal airway epithelial cells were used for transduction in gene-editing experiments (*n* = 3 donors). Following antibiotic selection, gene-edited basal epithelial cells were seeded onto 6.5 mm 24-well polyester Transwell inserts with 0.4 μm pores at 3.0 × 10^5^ cells/cm^2^ in ALI Expansion Media supplemented with Y-27632, and were air-lifted upon the development of an intact monolayer. Cultures were differentiated using PneumaCult ALI (PC-ALI) differentiation media (StemCell Technologies), and IL-13 stimulations were performed as described above.

### SDS-PAGE/western blot and dot blot analysis

ALI cultures were mock, or IL-13 stimulated in triplicate, as described above, and warm PBS was added to the apical chamber of each culture for 10 min at 37 °C prior to harvest and pooling. For SDS-PAGE apical wash samples were denatured at 94 °C for 7 min in Laemmli buffer loaded onto a BioRad TGX 4-20% SDS-PAGE gel at 150 V for 1 hour, and transferred to nitrocellulose at 90 V for 2 hours. For dot blots, 2 μl of each apical wash sample was applied to a nitrocellulose membrane and allowed to air dry. For all Western blots, membranes were blocked with 4% milk/0.1% Tween-20/TBS buffer and immunoblots were conducted using either rabbit anti-ITLN-1 (1:200; AbCam ab118232) in 4% milk/0.1% Tween-20/TBS overnight at 4 °C, or mouse anti-MUC5AC (1:1000; Fisher Scientific MA1-21907) in 4% milk/TBS/0.1% Tween-20 for 1 hour at 4 °C. Blots were probed with goat anti-rabbit (1:10,000; BioRad #170-6515) or goat anti-mouse (1:10,000; BioRad #170-6516) HRP-conjugated antibodies for 1 hour and incubated with ClarityMax Western ECL Blotting Substrate for chemiluminescent detection of protein bands. Images were captured on a BioRad ChemiDoc and band intensity densitometry was measured using ImageJ v1.53a.

### Mucociliary movement analysis using particle tracking

Control (scrb) and *ITLN1* KO ALI cultures described above were treated with BSA or IL-13 prior to particle tracking assays. Each insert was placed onto a plastic petri dish, and 20ul of Cy5-labeled 5 um beads (Bangs Laboratory, Inc; 1:500 beads diluted in ALI culture media) were added to the apical chamber of each culture using a P20 pipet. For each sample, 10 sec videos were captured on 4x magnification over 6 fields of view per culture insert, using triplicate culture inserts per sample treatment (BSA and IL-13) from all 3 edited donors (*n* = 18 total videos per donor-treatment pair; 3 inserts × 6 videos) using the Echo R4 microscope and QuickTime v10.5. Imaging was repeated for these same inserts after each of the following wash conditions: (1) No Wash, to capture the native MCM after 10d IL-13 stimulations, (2) PBS wash (10 min at 37 °C), aimed at hydrating and removing soluble mucus material, (3) PBS-DTT wash (10 mM DTT in PBS for 10 min at 37 °C) aimed at hydrating and denaturing the mucus layer, and (4) ATP + PBS-DTT wash (100 uM ATP in PBS in apical side of ALI cultures for 1 hour at 37 °C followed by 10 mM DTT wash) aimed at inducing secretion of intracellular mucus from mock and IL-13 stimulated cultures, followed by PBS-DTT wash aimed at hydration and denaturation of the ATP-induced mucus secretions. MOV-formatted videos were converted to greyscale and then imported into the Trackpy Python package (v0.4.1) for particle tracking analysis^41^. Briefly, particles were detected within frames (trackpy.batch function with diameter = 21 and minmass = 10) and then linked across frames into movement trajectories (tp.link_df function with search_range = 10 and memory = 10), where we removed particles not present in at least 25% of frames. For each video, based on an average of 305 detectable particles per video, we calculated the geometric mean of both total Euclidean displacement of each particle and total particle speed, as calculated by total particle displacement divided by the number of frames between first and last observance of a particle. We then estimated and tested the extent that the log of mean particle displacement or speed differed based on treatment (BSA control versus IL-13), *ITLN1* KO status (mock-targeted versus *ITLN1* KO), or their interaction for each wash regime (no wash, PBS wash, PBS-DTT wash, ATP + PBS-DTT wash) using a mixed model, where we included a random intercept for each donor and insert. Mixed models were run using the lmerTest R package v3.1.3^42^ and *p* values were calculated based on Satterthwaite-approximated degrees of freedom. The independently replicated particle tracking experiment was performed on the same 3 gene edited donors as used above, following re-differentiation at ALI, using a slightly different design as described below. For each sample, 10 sec videos were captured on 4x magnification over 4 fields of view per culture insert, using duplicate culture inserts per sample treatment (BSA and IL-13) from all 3 edited donors (*n* = average of 8 total videos per donor-treatment pair). Imaging was repeated for these same inserts after each of the following wash conditions: (1) No Wash, to capture the native MCM after 10d IL-13 stimulations, (2) PBS wash (10 min at 37 °C), aimed at hydrating and removing soluble mucus material, and (3) PBS-DTT wash (10 mM DTT in PBS for 10 min at 37 °C) aimed at hydrating and denaturing the mucus layer. Quantification of total displacement, particle speed, and all associated analyses were performed similar to that described above.

### Measurement of ciliary beat frequency

Using tracheal ALI cultures from control or *ITLN1*-targeted CRISPR-Cas9 gene editing experiments performed on 3 donors, triplicate ALI culture inserts were stimulated with BSA or IL-13 as described above, and were used to measure CBF to assess functional response to IL-13 in these epithelia. ALI cultures were first washed once in 37 °C PBS for 10 min, after which the wash was removed and cultures were imaged by placing the plastic insert in a glass-bottom Petri dish with ALI medium. Digital high-speed videos were recorded on 15 different fields of view across the outer perimeter edge of the sample, (FOV = 1280 × 1024 px, 1 px = 0.1625 μm), under bright-field illumination with DIC optics and at a sampling frequency of 170 fps using a Nikon Eclipse Ti-E inverted microscope (Nikon Instruments, Japan) with a 40x objective (Plan Apo λ ×40, N.A. 0.75, Nikon) fitted with an Andor Zyla sCMOS camera (Andor Technology Ltd, Belfast, United Kingdom). Epithelial cultures were imaged using a LiveCell^TM^ stage top incubator (Pathology Devides, Inc., San Diego, CA), where temperature, CO_2_, and humidity were continuously monitored and maintained at values of 37 °C, 5%, and 90%, respectively. Any videos that showed drifting or duplicated areas of analysis were not included in the analyses. CBF was quantified using Differential Dynamic Microscopy, as previously described^43,44^. Briefly, each field of view was first divided into tiles of 64 × 64 px (~10 μm per side). The distribution of CBF in the field of view was then measured by running DDM on the tiles that showed motion and fitting its output with an empirical oscillating function (the Image Structure Function). The CBF distributions for each treatment were then built by pooling the values of CBF (in units of Hz) measured on the active tiles of all the fields of view imaged across the samples. Total data points are listed below and were collected across triplicate culture inserts per treatment, editing condition, and donor: scrb control:BSA = 30,899; scrb control:IL-13 = 24,786, *ITLN1* KO:BSA = 25,533, *ITLN1* KO:IL-13 = 24,964. We then estimated and tested the extent that CBF differed based on treatment (BSA control versus IL-13), *ITLN1* KO status (mock-targeted versus *ITLN1* KO), or their interaction using a mixed model, where we included a random intercept for each donor and insert. Mixed models were run using the lmerTest R package and *p* values were calculated based on Satterthwaite-approximated degrees of freedom. Video capture for CBF analysis in the validation experiment differed slightly, as described below. Data were captured from 2 of the cultured and differentiated donors, as used in the previous experiment, and underwent either BSA (mock) or IL-13 stimulation. Videos were collected from 1 insert from each stimulation condition per donor for the scrb controls, and 2 inserts from each stimulation condition per donor for the ITLN1 KO samples. The digital high-speed videos were recorded on 5 different fields of view across the sample (FOV = 1920 × 1024 px, 1 px = 0.146 µm), under bright-field illumination and at a sampling frequency of 150 fps using a Nikon Eclipse Ti-E inverted microscope (Nikon Instruments, Japan) with a 40x objective (Plan Apo λ ×40, N.A. 0.95, Nikon) fitted with a Grasshopper®3 GS3-U3-23S6M-C CMOS camera (FLIR Integrated Imaging Solutions GmbH, Germany). Epithelial cultures were imaged in a custom-made chamber where temperature, CO_2_ and humidity were continuously monitored and maintained at values of 37 °C, 5%, and 90%, respectively. CBF was quantified and analyzed in a similar manner as described above resulting in total data points listed here: scrb control:BSA = 3061; scrb control:IL-13 = 1640, *ITLN1* KO:BSA = 5519, *ITLN1* KO:IL-13 = 4887.

### Quantitative PCR gene expression analysis

Experiments using tracheal airway ALI epithelial cultures to validate the gene expression effects of *ITLN1* rs4656959 (*n* = 5 patient samples per genotype), and from tracheal CRISPR gene editing experiments were harvested in RNA/DNA Lysis Buffer supplemented with 40 mM dithiothreitol (DTT). Triplicate culture inserts per condition were pooled, and lysates were extracted using the Zymo MiniRNA Kit (Zymo Research) and cDNA was synthesized using the Maxima First Strand cDNA Synthesis Kit for qRT-PCR (Thermo Scientific). For qPCR analysis, cDNA samples were amplified and analyzed using a QuantStudio 6 Flex Real-Time PCR System (Life Technologies). Assays were run using Brilliant III Master Mix (Agilent) using the following cycling conditions: 95 °C for 3 minutes, followed by 40 cycles of 95 °C 10 seconds and 60 °C for 30 seconds using PrimeTime qPCR 5′ Nuclease probe assays for *GusB* (Hs.PT.51.2648420; IDT), *ITLN1* (Hs.PT.58.39839277; IDT), *MUC5AC* (Hs01365600_g1; Thermo), *MUC5B* (Hs.PT.51.5074152.gs; IDT), and *SCGB1A1* (Hs.PT.58.1190800; IDT). Expression was normalized to the airway epithelial housekeeping gene (*GUSB)*.

### Human mucin and peptide binding assays

Human purified mucins were obtained from asthmatic-induced sputum by purification of high molecular weight proteins, as described previously^13,45^. Five asthmatic donors were used to collect and purify mucins, and samples were pooled together for the mucin-binding assays. Human purified mucins, MUC5AC C-terminal recombinant peptide (AA5568-AA5654, Mybiosource, #MBS2009104) or MUC5B C-terminal recombinant peptide (AA5653-AA5762, LifeSpan Biosciences, G12612), were coated on a NUNC Maxisorp plate overnight at 4 °C at the concentration of 20μg/mL in carbonate-bicarbonate buffer, pH = 9.6. Plates were then blocked with blocking buffer (TBS, CaCl_3_ 3 mM, BSA 3%, Tween-20 0.05%) for 2 hours at room temperature. FLAG-tagged recombinant ITLN-1 (Sigma, SRP8047) was incubated for 2 hours at 10μg/mL in binding buffer B (TBS, 3 mM CaCl_3_, 1% BSA, 0.05% Tween-20, 50 mM EDTA) for purified mucin binding or binding buffer A (TBS, 3 mM CaCl_3_, 1% BSA, 0.05% Tween-20) for recombinant peptide binding. For binding inhibition assays, heparin (150μg/mL, Sigma, H3393), dextran sulfate (150μg/mL, Sigma D8906), or galactose (100 mM, Sigma G0750) were added to the binding buffer. To detect ITLN-1 binding to coated mucins, an anti-FLAG HRP-conjugated antibody (ThermoFisher, MA1-91878-HRP) was incubated for 1 hour. The signal was detected using TMB substrate (SeraCare, 5120-0047), and the absorbance was read with a plate reader at 450 nm. For binding assays with heparin, biotin-conjugated heparin was used (Millipore, 375054) at 10μg/mL. To detect heparin binding to coated mucins, plates were incubated with ExtrAvidin-alkaline phosphatase (Sigma, E2636) for 1 hour. The signal was revealed using the phosphatase substrate (Sigma, S0942), and the absorbance was read with a plate reader at 405 nm. The control conditions for ITLN-1 and heparin assays correspond to binding buffer only or to biotinylated albumin 10μg/mL, respectively.

### scRNA-seq analysis of bronchial brushings

Bronchial cells were dissociated from the bronchial airway brushes from 2 asthmatic children using *Bacillus licheniformis* cold active protease (10 mg/ml; Sigma P5380-1G), EDTA (0.5 mM), and EGTA (0.5 mM) at 4 °C with vortex mixing, followed by enzyme neutralization with FBS. Red blood cell lysis was performed and cells were washed twice in 0.04% BSA/PBS. Targeted capture of 8000 cells was performed using the 10X Genomics Chromium Next GEM Single Cell 3′ reagent kit chemistry. Sample capture, cDNA synthesis, and library preparation was performed following 10X Genomics Chromium Next GEM Single Cell 3′ chemistry (CG000204 Rev D), and barcoded samples were pooled and sequenced on an Illumina NovaSeq 6000. Sequencing reads were processed with Cell Ranger (version 4) to produce a gene UMI count matrix. We required cells to have at least 500 genes expressed and 1000 UMIs. Additionally, we removed cells with greater than 30% MT reads, resulting in 11,515 remaining cells to use for clustering analysis. Integration of the two bronchial brushing datasets was carried out with the SCtransform integration approach implemented in Seurat v4.0 using the top 30 canonical correlates derived from 5000 most variable genes across the two datasets. We then carried out PCA on the integrated dataset and used the top 30 PC dimensions for UMAP visualization and to perform SNN clustering with the Leiden algorithm (resolution = 0.3, k.param = 20)^46^, resulting in 17 cell clusters. These cell clusters were then assigned a cell type label based on the list of the marker genes produced with Seurat’s FindMarkers() function (Supplementary Data 2).

### RNA-seq analysis of IL13-stimulated bronchial ALI cultures

Ion AmpliSeq Transcriptome Human Gene Expression RNA-sequencing (RNA-seq) libraries (ThermoFisher) were constructed with 100 ng of RNA per sample. Barcoded libraries were pooled and sequenced on the Ion Torrent Proton sequencer using P1 chips. Sequencing reads were mapped to AmpliSeq transcriptome target regions with the torrent mapping alignment program and quantified with the Ion Torrent AmpliSeq RNA plugin using the unique mapping option. Duplicated sequences were removed from the FASTA file, and incorrect amplicon locations were corrected^7^.

### Whole-transcriptome analysis of the GALA II study nasal brushes and tracheal ALI cultures

Primary nasal airway epithelial brushes were obtained from Puerto Rican children recruited as a part of the Genes-environments and Admixture in Latino Americans II (GALA II) childhood asthma study and placed into RLT Plus lysis buffer supplemented with 40 mM DTT for RNA and DNA extraction using the AllPrep DNA/RNA MiniPrep Kit (Qiagen). Tracheal ALI cultures with or without 48 hr IL-13 stimulation were harvested for RNA extraction and stored at -80 °C prior to analysis. RNA normalization, library construction using KAPA Stranded mRNA-Seq Kit with KAPA mRNA Capture Beads (KAPA Biosystems), and library pooling for a total of 695 patients were all performed on the Beckman Coulter FX^P^ automation system. Briefly, RNA samples were randomized over the normalization plate in alternating control and asthmatic conditions at 250 ng per well. Illumina Dual-Index adapters (Integrated DNA Technologies) were used to barcode libraries using 12 cycles of amplification. Samples were pooled using equal concentrations of six libraries/pool and pair-end sequenced with Illumina HiSeq® 2500 System sequencing. Sequencing reads were trimmed with Skewer (--end-quality 15, --mean-quality 25, --min 30)^47^. Trimmed reads were aligned and mapped to human reference genome GRCh38 with GSNAP^48^. Transcript quantification of these aligned reads on iGenomes GTF file was conducted with HTSeq to generate a gene count matrix^49^. Additionally, Kallisto was used to generate Transcript per Million (TPM) values for all the samples^50^. A total of 17,039 genes with TPM > 0.1 and read counts >6 for at least 20% of the samples were subjected to downstream eQTL analysis. Differential expression analysis of *ITLN1* expression between genotype groups stratified by type 2 status was performed with DESeq2^51^.

### Genotype analysis for GALA II study

Blood samples were collected from participants in the Genes-environments and Admixture in Latino Americans study (GALA II) asthma study, and a total of 681 were included in this analysis. The genotype and phenotype information analyzed in this manuscript is publicly available via dbGAP (dbGAP accession numbers: phs001274.v1.p1, phs001180.v1.p1, and phs00921.v1.p1). Samples were genotyped with Affymetrix Axiom LAT 1 (World Array 4) and LAT plus HLA genome-wide arrays (Affymetrix, Santa Clara, CA). SNPs were removed if they failed manufacturer’s quality control, had genotyping call rates below 95%, and/or had a deviation from Hardy–Weinberg equilibrium (*p* < 10^−6^) within controls. Samples were filtered if there was a discrepancy between genetic sex and reported gender. When cryptic relatedness (PI_HAT > 0.3) was detected for a pair of samples, one of them was removed.

### GALA II *ITLN1* eQTL analysis

Expression quantitative trait loci (eQTL) analysis was performed following a pipeline similar to the Genotype-Tissue Expression (GTEx) V7 protocol^52^ using paired genotype-gene expression data from 681 GALA II donors. After initial filtering on minor allele frequency (>0.01), minor allele sample count (>10), a total of 8432 SNPs within 1 Mb of *ITLN1* TSS were tested for association with *ITLN1* gene expression level. *ITLN1* gene expression was TMM-normalized with edgeR followed by inverse normalization^53,54^. Probabilistic Estimation of Expression Residuals (PEER) factors were computed with R package PEER^55^. Admixture factors were estimated with the software Admixture^56^. *ITLN1* eQTL mapping was performed with FastQTL^57^, and adjusted for age, gender, body mass index (BMI), asthma status, 60 PEER factors, and four admixture factors. A stepwise regression approach implemented in QTLtools^58^ was used to identify independent *ITLN1* eQTLs.

### Sputum induction and processing

Participants inhaled nebulized 3% saline through a mouthpiece for 12 minutes, and interrupted inhalation at 2-minute intervals to spit saliva into a saliva cup and induce sputum into a sputum cup^59^. Saliva was discarded, and induced sputum was processed. A 10% solution of Sputolysin (EMD Millipore) was added at a 1:1 g/ml (sputum weight/Sputolysin) ratio to the induced sputum, mixed with a serologic pipette, and placed in a 37 °C shaking water bath for 15 minutes. Samples were mixed at intervals; total and differential cell counts were determined. The sample was then centrifuged at 4 °C at 900 × *g* for 10 minutes. The cell pellet was resuspended in 1 ml of Qiagen RNAprotect Saliva Reagent (Qiagen). Cell pellets were stored at −80 °C, and all RNA was shipped to the UCSF Sputum Core for RNA extraction using the RNeasy Qiagen kit (Qiagen), as previously described^60,61^. RNA concentration and quality were measured with the Agilent 2100 bioanalyzer, and samples with an RNA integrity number <5 were considered degraded and excluded from analysis.

### SARP study genotyping

DNA from participants enrolled in the SARP3 study was genotyped using the Illumina Multi-Ethnic Global BeadChip (MEGA) Chip as previously described^62^.

### Sputum whole-transcriptome RNA sequencing

KAPA mRNA HyperPrep (Roche) whole-transcriptome libraries were constructed with 20 ng RNA input per sample, barcoded with Illumina Dual Index Adapters, and amplified for 16 cycles. Completed libraries were pooled together by concentration and sequenced using the Illumina NovaSeq 6000 system. Raw sequencing reads were trimmed using skewer^47^ with end-quality = 15, mean-quality = 25, min = 30. Trimmed reads were then aligned to the human reference genome, GRCh38, using HiSat2 with default parameters^63^. Gene quantification was performed with htseq-count using the GRCh38 ensemble v84 gene transcript model^49^. Variance stabilization transformation implemented in DESeq2 was then performed on the raw gene count matrix to create a variance-stabilized gene expression matrix suitable for downstream analyses. Differential expression analysis of *ITLN1* expression between genotype groups were performed with DESeq2^51^.

### Analysis of mucus plugging scores

Airway lumen mucus plugs were identified in participants enrolled in the SARP using multidetector computed tomography (MDCT) scans, across sequential transverse CT slices, and were formally quantified as previously described^8^. CT scans were independently scored by radiologists using a developed visual scoring system, and the mucus plug scores were then averaged. To test for association between rs4656959 genotype and mucus plug scores, we used the negative binomial mixed model implemented in SAS (Cary, NC) software v9.04 PROC GLIMMIX (METHOD = RSPL; DDFM = KENWARDROGER2), with subjectID as random effect and adjusting for age, gender, and two admixture factors. Prior to model fitting, mucus plug scores were converted into integers using *ceiling* function in *R*.

### Weighted gene coexpression network analysis

To capture the heterogeneity of transcriptomic profiles from cultured airway epithelial, nasal airway epithelial, and airway sputum, we performed weighted gene coexpression network analysis (WGCNA) on the variance-stabilized expression matrix from each respective sample type. The gene networks from the IL-13-stimulated HBEC ALI cultures were constructed on 15,152 genes using the following parameters: softPower = 10, deepSplit = 2, minClusterSize = 40, and PAM = F, resulting in 18 gene networks. The construction of gene coexpression networks from the GALA II nasal samples has been described elsewhere^4^. Briefly, WGCNA was run on 17,473 expressed genes with softPower = 9, minClusterSize = 20, deepSplit = 2, and PAM = T. Similarly, WGCNA was performed on 18,197 expressed genes from the SARP sputum samples with softPower = 12, minClusterSize = 10, deepSplit = 1, and PAM = T. To classify type 2 status, first, we hierarchically clustered longitudinal SARP samples using ward.D2 and the Pearson correlation distance metric using the normalized expression of type 2 module genes (Supplementary Data 4) and used the first split of the constructed dendrogram to classify samples into type 2-low and type 2-high groups. A participant was classified as type 2-high if any of its associated longitudinal samples belonged to the type 2-high group, otherwise they were classified as type 2-low.

### Reporting summary

Further information on research design is available in the Nature Portfolio Reporting Summary linked to this article.

### Supplementary information

Supplementary Information

Description of Additional Supplementary Files

Supplementary Data 1

Supplementary Data 2

Supplementary Data 3

Supplementary Data 4

Supplementary Data 5

Reporting Summary

### Source data

Source Data

## Data Availability

Gene lists associated with the main figure and Supplementary Fig. panels can be found in the Supplementary Data provided with this manuscript. GALA II RNA-seq data used in this study have been previously deposited in the National Center for Biotechnology Information (NCBI)/Gene Expression Omnibus (GEO) GSE152004. Data used as part of the Human Lung Atlas for scRNA-seq analyses can be found in the Supplementary Data and at https://hlca.ds.czbiohub.org/ as reported in the cited manuscript by Travaglini et al.^17^. The raw and processed scRNA-seq data used in this study have been deposited in the NCBI/GEO with accession number GSE254127. The processed protein and RNA-seq gene expression data generated from in vitro experiments can be found at the github repository [https://github.com/seiboldlab/ITLN1_paper]. Data underlying manuscript figures involving the SARP cohort are provided in the github repository. Source data are provided with this paper.

## Code availability

The code used to produce figures and perform analyses in the study can be found on GitHub [https://github.com/seiboldlab/ITLN1_paper].

## References

[1] Fahy JV (2015). Type 2 inflammation in asthma–present in most, absent in many. Nat. Rev. Immunol..

[2] Wesolowska-Andersen A, Seibold MA (2015). Airway molecular endotypes of asthma: dissecting the heterogeneity. Curr. Opin. Allergy Clin. Immunol..

[3] Woodruff PG (2009). T-helper type 2-driven inflammation defines major subphenotypes of asthma. Am. J. Respir. Crit. Care Med..

[4] Sajuthi SP (2020). Type 2 and interferon inflammation regulate SARS-CoV-2 entry factor expression in the airway epithelium. Nat. Commun..

[5] Jackson ND (2020). Single-cell and population transcriptomics reveal pan-epithelial remodeling in type 2-high asthma. Cell Rep..

[6] Peters MC (2019). A transcriptomic method to determine airway immune dysfunction in T2-high and T2-low asthma. Am. J. Respir. Crit. Care Med.

[7] Poole A (2014). Dissecting childhood asthma with nasal transcriptomics distinguishes subphenotypes of disease. J. Allergy Clin. Immunol..

[8] Dunican EM (2018). Mucus plugs in patients with asthma linked to eosinophilia and airflow obstruction. J. Clin. Invest..

[9] Bonser LR, Zlock L, Finkbeiner W, Erle DJ (2016). Epithelial tethering of MUC5AC-rich mucus impairs mucociliary transport in asthma. J. Clin. Invest..

[10] Lachowicz-Scroggins ME (2016). Abnormalities in MUC5AC and MUC5B protein in airway mucus in asthma. Am. J. Respir. Crit. Care Med..

[11] Wesener DA (2015). Recognition of microbial glycans by human intelectin-1. Nat. Struct. Mol. Biol..

[12] Tsuji S (2001). Human intelectin is a novel soluble lectin that recognizes galactofuranose in carbohydrate chains of bacterial cell wall. J. Biol. Chem..

[13] Kerr SC (2014). Intelectin-1 is a prominent protein constituent of pathologic mucus associated with eosinophilic airway inflammation in asthma. Am. J. Respir. Crit. Care Med..

[14] Pemberton AD, Rose-Zerilli MJ, Holloway JW, Gray RD, Holgate ST (2008). A single-nucleotide polymorphism in intelectin 1 is associated with increased asthma risk. J. Allergy Clin. Immunol..

[15] Barrett JC (2008). Genome-wide association defines more than 30 distinct susceptibility loci for Crohn’s disease. Nat. Genet..

[16] Franke A (2010). Genome-wide meta-analysis increases to 71 the number of confirmed Crohn’s disease susceptibility loci. Nat. Genet..

[17] Travaglini KJ (2020). A molecular cell atlas of the human lung from single-cell RNA sequencing. Nature.

[18] Xu G, Forstner GG, Forstner JF (1996). Interaction of heparin with synthetic peptides corresponding to the C-terminal domain of intestinal mucins. Glycoconj. J..

[19] Xu G, Bell SL, McCool D, Forstner JF (2000). The cationic C-terminus of rat Muc2 facilitates dimer formation post translationally and is subsequently removed by furin. Eur. J. Biochem..

[20] Sajuthi, S. P. et al. Type 2 and interferon inflammation strongly regulate SARS-CoV-2 related gene expression in the airway epithelium. *bioRxiv* https://www.biorxiv.org/content/10.1101/2020.04.09.034454v1 (2020). 10.1038/s41467-020-18781-2 PMC7550582 33046696

[21] Sajuthi SP (2022). Nasal airway transcriptome-wide association study of asthma reveals genetically driven mucus pathobiology. Nat. Commun..

[22] Kesimer M (2017). Airway mucin concentration as a marker of chronic bronchitis. N. Engl. J. Med..

[23] Radicioni G (2021). Airway mucin MUC5AC and MUC5B concentrations and the initiation and progression of chronic obstructive pulmonary disease: an analysis of the SPIROMICS cohort. Lancet Respir. Med..

[24] Nonnecke EB (2021). Human intelectin-1 (ITLN1) genetic variation and intestinal expression. Sci. Rep..

[25] Gu N (2010). Intelectin is required for IL-13-induced monocyte chemotactic protein-1 and -3 expression in lung epithelial cells and promotes allergic airway inflammation. Am. J. Physiol. Lung Cell Mol. Physiol..

[26] Kuperman DA (2005). Dissecting asthma using focused transgenic modeling and functional genomics. J. Allergy Clin. Immunol..

[27] Watanabe T (2017). Expression of intelectin-1 in bronchial epithelial cells of asthma is correlated with T-helper 2 (Type-2) related parameters and its function. Allergy Asthma Clin. Immunol..

[28] Yuan S (2015). Oxidation increases mucin polymer cross-links to stiffen airway mucus gels. Sci. Transl. Med..

[29] Tang M (2022). Mucus plugs persist in asthma, and changes in mucus plugs associate with changes in airflow over time. Am. J. Respir. Crit. Care Med..

[30] Neophytou AM (2016). Air pollution and lung function in minority youth with asthma in the GALA II (genes-environments and admixture in Latino Americans) and SAGE II (Study of African Americans, Asthma, Genes, and Environments) studies. Am. J. Respir. Crit. Care Med..

[31] Nishimura KK (2013). Early-life air pollution and asthma risk in minority children. The GALA II and SAGE II studies. Am. J. Respir. Crit. Care Med..

[32] Yamaya M, Finkbeiner WE, Chun SY, Widdicombe JH (1992). Differentiated structure and function of cultures from human tracheal epithelium. Am. J. Physiol..

[33] Reynolds SD (2016). Airway progenitor clone formation is enhanced by Y-27632-dependent changes in the transcriptome. Am. J. Respir. Cell Mol. Biol..

[34] Everman JL, Rios C, Seibold MA (2018). Utilization of air-liquid interface cultures as an in vitro model to assess primary airway epithelial cell responses to the type 2 cytokine interleukin-13. Methods Mol. Biol..

[35] Kotas ME (2022). IL-13-programmed airway tuft cells produce PGE2, which promotes CFTR-dependent mucociliary function. JCI Insight.

[36] Yu Q (2020). Benchmarking the orbitrap tribrid eclipse for next generation multiplexed proteomics. Anal. Chem..

[37] Newman AM (2019). Determining cell type abundance and expression from bulk tissues with digital cytometry. Nat. Biotechnol..

[38] Chu HW (2015). CRISPR-Cas9-mediated gene knockout in primary human airway epithelial cells reveals a proinflammatory role for MUC18. Gene Ther..

[39] Everman JL, Rios C, Seibold MA (2018). Primary airway epithelial cell gene editing using CRISPR-Cas9. Methods Mol. Biol..

[40] Conant D (2022). Inference of CRISPR edits from sanger trace data. CRISPR J..

[41] Allan D. B. et al. soft-matter/trackpy: Trackpy v0.5.0 (v0.5.0) https://zenodo.org/records/4682814 (2021).

[42] Kuznetsova A, Brockhoff PB, Christensen RHB (2017). lmerTest package: tests in linear mixed effects models. J. Stat. Softw..

[43] Chioccioli M (2019). Quantitative high-speed video profiling discriminates between DNAH11 and HYDIN variants of primary ciliary dyskinesia. Am. J. Respir. Crit. Care Med..

[44] Feriani L (2017). Assessing the collective dynamics of motile cilia in cultures of human airway cells by multiscale DDM. Biophys. J..

[45] Royle L (2008). Glycan structures of ocular surface mucins in man, rabbit and dog display species differences. Glycoconj. J..

[46] Traag VA, Waltman L, van Eck NJ (2019). From Louvain to Leiden: guaranteeing well-connected communities. Sci. Rep..

[47] Jiang H, Lei R, Ding SW, Zhu S (2014). Skewer: a fast and accurate adapter trimmer for next-generation sequencing paired-end reads. BMC Bioinformatics.

[48] Wu TD, Nacu S (2010). Fast and SNP-tolerant detection of complex variants and splicing in short reads. Bioinformatics.

[49] Anders S, Pyl PT, Huber W (2015). HTSeq–a Python framework to work with high-throughput sequencing data. Bioinformatics.

[50] Bray NL, Pimentel H, Melsted P, Pachter L (2016). Near-optimal probabilistic RNA-seq quantification. Nat. Biotechnol..

[51] Love MI, Huber W, Anders S (2014). Moderated estimation of fold change and dispersion for RNA-seq data with DESeq2. Genome Biol..

[52] Consortium GT LD (2017). Coordinating center-analysis working G, statistical methods groups-analysis working G, enhancing Gg, Fund NIHC, et al. Genetic effects on gene expression across human tissues. Nature.

[53] Robinson MD, McCarthy DJ, Smyth GK (2010). edgeR: a Bioconductor package for differential expression analysis of digital gene expression data. Bioinformatics.

[54] McCarthy DJ, Chen Y, Smyth GK (2012). Differential expression analysis of multifactor RNA-Seq experiments with respect to biological variation. Nucleic Acids Res..

[55] Stegle O, Parts L, Piipari M, Winn J, Durbin R (2012). Using probabilistic estimation of expression residuals (PEER) to obtain increased power and interpretability of gene expression analyses. Nat. Protoc..

[56] Alexander DH, Novembre J, Lange K (2009). Fast model-based estimation of ancestry in unrelated individuals. Genome Res..

[57] Ongen H, Buil A, Brown AA, Dermitzakis ET, Delaneau O (2016). Fast and efficient QTL mapper for thousands of molecular phenotypes. Bioinformatics.

[58] Delaneau O (2017). A complete tool set for molecular QTL discovery and analysis. Nat. Commun..

[59] Gershman NH, Wong HH, Liu JT, Mahlmeister MJ, Fahy JV (1996). Comparison of two methods of collecting induced sputum in asthmatic subjects. Eur. Respir. J..

[60] Peters MC (2019). Refractory airway type 2 inflammation in a large subgroup of asthmatic patients treated with inhaled corticosteroids. J. Allergy Clin. Immunol..

[61] Peters MC (2014). Measures of gene expression in sputum cells can identify TH2-high and TH2-low subtypes of asthma. J. Allergy Clin. Immunol..

[62] Cardet JC (2022). Clinical and molecular implications of RGS2 promoter genetic variation in severe asthma. J. Allergy Clin. Immunol..

[63] Kim D, Langmead B, Salzberg SL (2015). HISAT: a fast spliced aligner with low memory requirements. Nat. Methods.

