# Supplementary material for: A common polymorphism in the Intelectin-1 gene influences mucus plugging in severe asthma
Source: Nat Commun. 2024 May 9;15:3900. doi: 10.1038/s41467-024-48034-5 (PMC11082194; doi:10.1038/s41467-024-48034-5)
Supplement: Supplementary file 1 — Supplementary Information [file 41467_2024_48034_MOESM1_ESM.pdf]

Supplemental Figures

SFigure 1

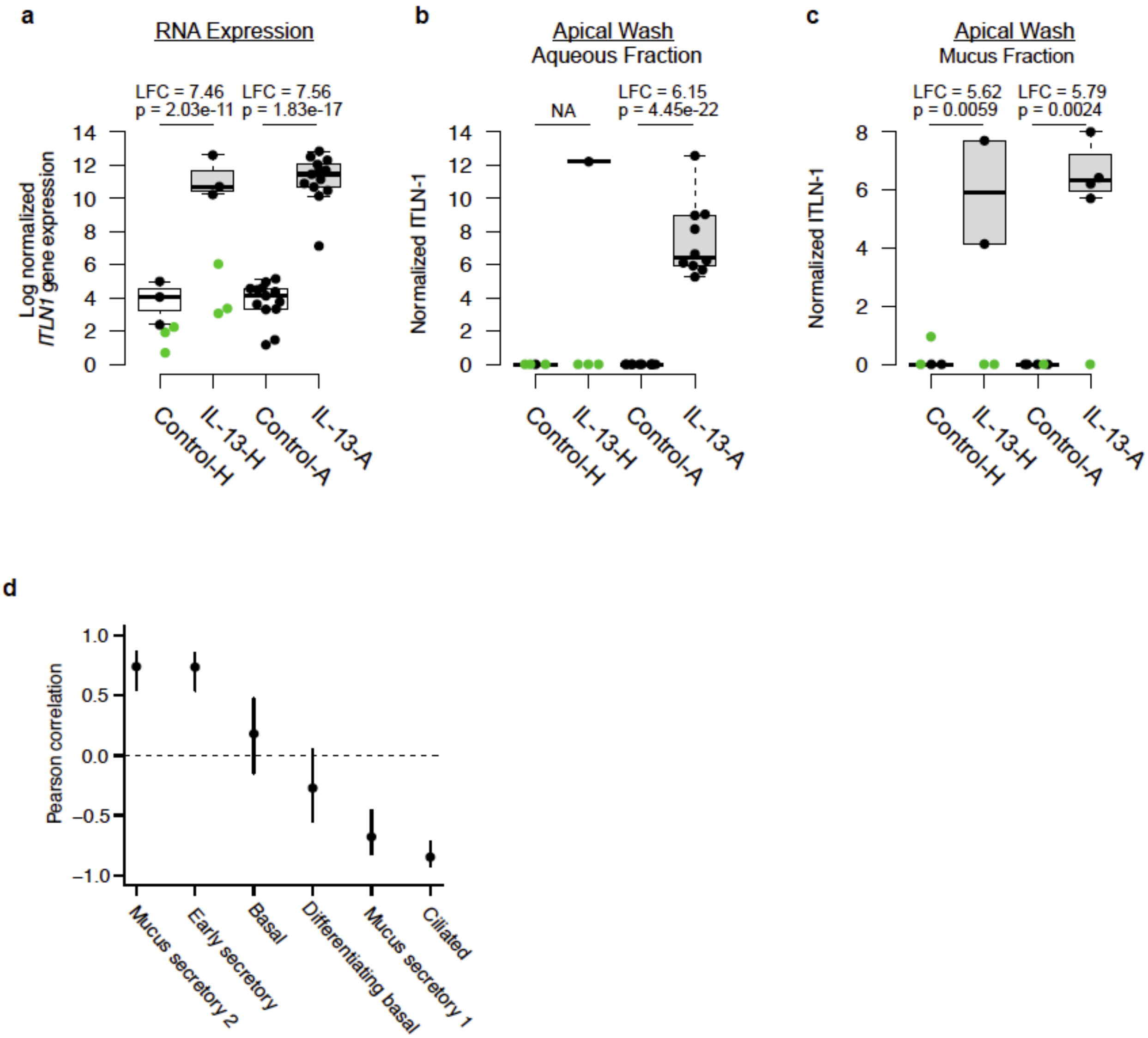

SFigure 1: IL-13 induction of ITLN-1 in mucociliary cultures derived from control and asthmatic airway brushes

(a) Log normalized *ITLN1* gene expression in BSA (control) and IL-13 treated ALI cultures generated from healthy (H; n=6) and asthmatic (A; n=13) participants. Boxplots, log fold change (LFC) values, and statistics are representative of data from donors with the *ITLN1*-expressing rs4656959 A/A or A/G genotype (H, n=3; A, n=13). Data points from donors with the *ITLN1* rs4656959 variant G/G genotype described in this manuscript (H, n=3) are excluded from the statistical analyses and boxplots of gene expression between healthy and asthmatic-derived ALI cultures, but are included in these plots as green data points for transparency of all the data shown in Figure 1b (n=19 total donors). FDR-adjusted two-sided p-value is based on a paired exact test (edgeR). Box centers = median, upper and lower box bounds = 1st and 3rd quartiles, whiskers extend from these bounds up to 1.5 x IQR (inter-quartile range). All data points are overlain.

(b) Normalized ITLN-1 peptides measured from independently collected apical aqueous ALI secretions following control and chronic IL-13 stimulation of mucociliary cultures generated from a subset of healthy (H, n=4) and asthmatic (A, n=10) donor cultures. Boxplots, log fold change (LFC) values, and statistics are representative of data from donors with the *ITLN1*-expressing rs4656959 A/A or A/G genotype (H, n=1; A, n=10). Data points from donors with the *ITLN1* rs4656959 variant G/G genotype (H, n=3) are excluded from the statistical analyses and boxplots of protein detection between healthy and asthmatic-derived ALI cultures, but are included in these plots as green data points for transparency of all the data shown in Figure 1f: Apical Washes-Aqueous Fraction (n=14 total donors). FDR-adjusted two-sided p-values are based on a paired exact test (edgeR). LFC = log fold change. Box plots are as in (a).

(c) Normalized ITLN-1 peptides measured from independently collected apical ALI mucus fractions following control and chronic IL-13 stimulation of mucociliary cultures generated from healthy (H, n=4) and asthmatic (A, n=5). Boxplots, log fold change (LFC) values, and statistics are representative of data from donors with the *ITLN1*-expressing rs4656959 A/A or A/G genotype (H, n=2; A, n=4). Data points from donors with the *ITLN1* rs4656959 G/G genotype (H, n=2; A, n=1) are excluded from the statistical analyses and boxplots of protein detection between healthy and asthmatic-derived ALI cultures, but are included in these plots as green data points for transparency of all the data shown in Figure 1f: Apical Washes-Mucus Fraction (n=9 total donors). FDR-adjusted two-sided p-values are based on a paired exact test (edgeR). LFC = log fold change. Box plots are as in (a).

(d) Pearson correlations between *ITLN1* network expression (see Fig. 1c) and cell type proportions in bronchial airway epithelial RNA-seq data from ALI cultures (n=19 donors), as imputed using CIBERSORTx based on six major epithelial cell types in scRNA-seq data from bronchial brushings (see Fig. 4c). Points give correlation coefficients and vertical lines give the confidence interval.

Source data are provided as a Source Data file.

SFigure 2

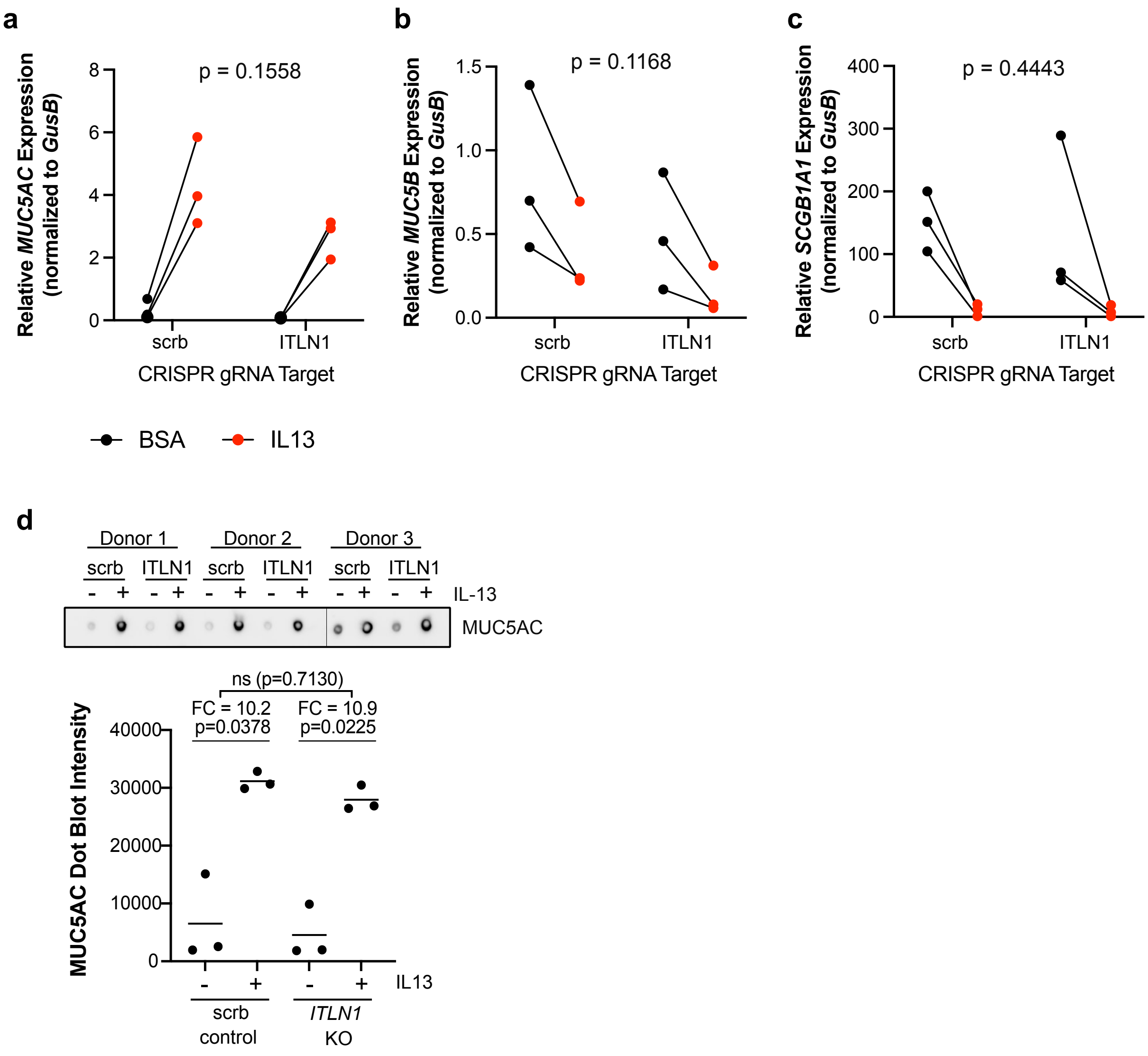

a
p = 0.1558
Relative MUC5AC Expression (normalized to GusB)
8
6
4
2
0
scrb
ITLN1
CRISPR gRNA Target
b
p = 0.1168
Relative MUC5B Expression (normalized to GusB)
1.5
1.0
0.5
0.0
scrb
ITLN1
CRISPR gRNA Target
c
p = 0.4443
Relative SCGB1A1 Expression (normalized to GusB)
400
300
200
100
0
scrb
ITLN1
CRISPR gRNA Target
BSA
IL13
d
Donor 1
Donor 2
Donor 3
scrb
ITLN1
IL-13
MUC5AC
ns (p=0.7130)
FC = 10.2
p=0.0378
FC = 10.9
p=0.0225
MUC5AC Dot Blot Intensity
40000
30000
20000
10000
0
IL13
scrb control
ITLN1 KO

SFigure 2: IL-13 response markers of gene-edited mucociliary cultures

(a-c) Normalized *MUC5AC, MUC5B,* and *SCGB1A1* expression measured by qPCR from tracheal mucociliary scrb- and ITLN1-edited ALI cultures following mock (BSA – black) or IL-13 (red) stimulation from 3 edited donors. Two-sided p-values calculated by paired Student's t-test based on comparisons between the calculated BSA:IL-13 fold-changes from each donor.

(d) Representative dot blot images of apical washes collected from 3 gene-edited donors following mock or IL-13 stimulation (top), and quantified MUC5AC abundance from all edited donors (bottom). Data plotted as individual values from each donor (n=3) with mean illustrated in each group; Two-sided p-values calculated using paired Student's t-test based on comparisons between BSA:IL13 changes in scrb and ITLN1 KO cultures, and based on BSA:IL13 fold changes for each donor between scrb and KO groups.

Source data are provided as a Source Data file.

SFigure 3

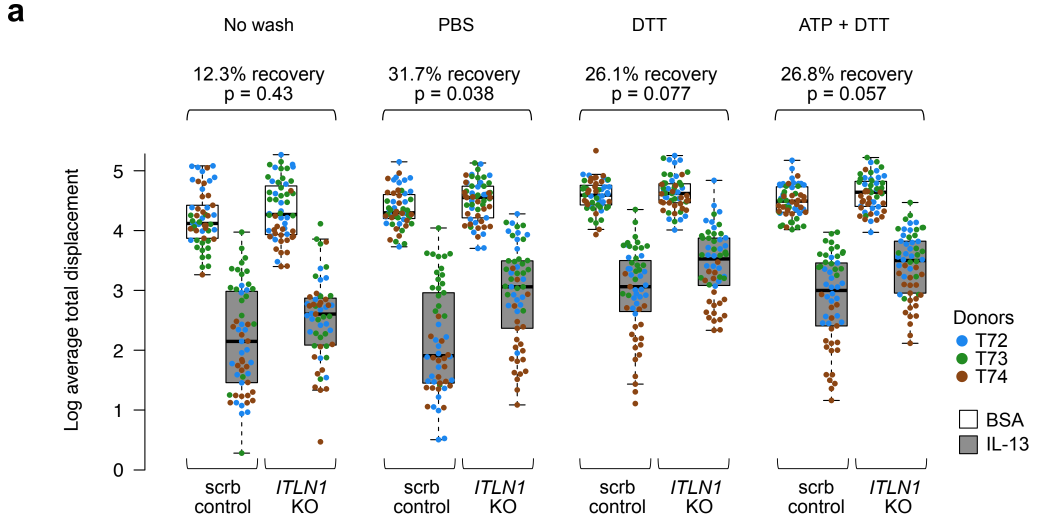
a
No wash
PBS
DTT
ATP + DTT
12.3% recovery
p = 0.43
31.7% recovery
p = 0.038
26.1% recovery
p = 0.077
26.8% recovery
p = 0.057
Log average total displacement
0
1
2
3
4
5
scrb control
ITLN1 KO
Donors
T72
T73
T74
BSA
IL-13

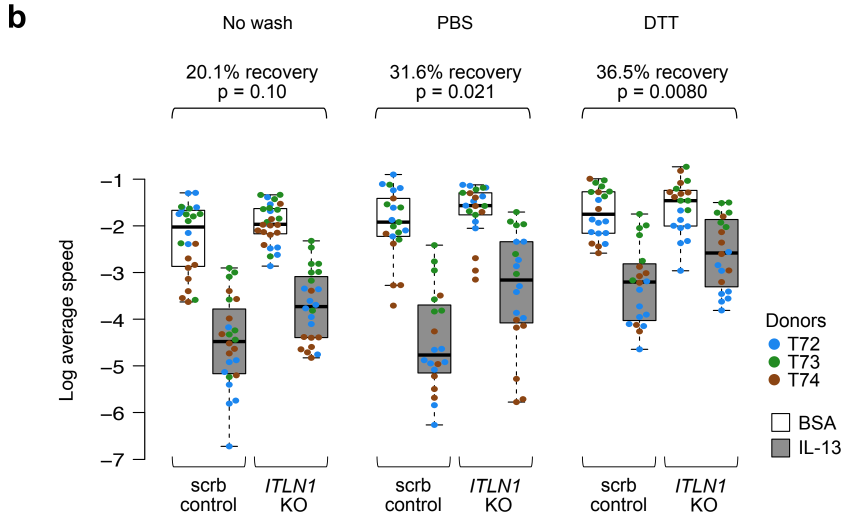
b
No wash
PBS
DTT
20.1% recovery
p = 0.10
31.6% recovery
p = 0.021
36.5% recovery
p = 0.0080
Log average speed
−1
−2
−3
−4
−5
−6
−7
scrb control
ITLN1 KO
Donors
T72
T73
T74
BSA
IL-13

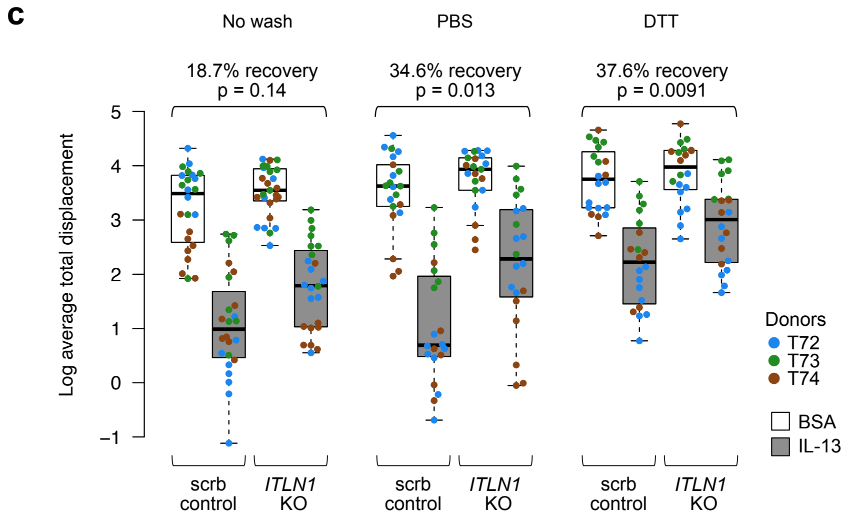
c
No wash
PBS
DTT
18.7% recovery
p = 0.14
34.6% recovery
p = 0.013
37.6% recovery
p = 0.0091
Log average total displacement
5
4
3
2
1
0
−1
scrb control
ITLN1 KO
Donors
T72
T73
T74
BSA
IL-13

SFigure 3: ITLN-1 contributes to IL-13-mediated decrease in epithelial mucociliary function (mucociliary movement)

(a) Box plots showing the log of average particle displacement within control edited (scrb) and *ITLN1* KO mucociliary cultures that were mock- (BSA, white boxes) or IL-13-stimulated (grey boxes), with replicate experiments carried out using no wash, PBS wash, PBS-DTT wash, and ATP + PBS-DTT wash regimes. Data values represent the log of average displacement of all measured particles within each video from the MCM assays, where there are an average of n=18 videos for each condition (2 KO statuses x 2 IL-13 treatments x 4 washes) per donor (~n=6 fields of view measured across n=3 individual ALI inserts from each of the n=3 edited donors – with each donor identified by a different color in the plot). The exact sample sizes per box, left to right are 54, 55, 56, 55, 55, 54, 58, 58, 55, 55, 55, 56, 56, 54, 55, 58. Above plots are given the estimated % recovery (and associated p-values) of particle displacement in IL-13-stimulated cultures relative to BSA cultures when in an *ITLN1* KO epithelium compared to the control (scrb) epithelium. Two-sided p-values are based on linear mixed model t-tests, with random effects for donor and insert, and use Satterthwaite approximation of degrees of freedom. Box centers = median, upper and lower box bounds = 1st and 3rd quartiles, whiskers extend from these bounds up to 1.5 x IQR (inter-quartile range). All data points are overlain.

(b-c) Independently performed particle displacement experiment using the same 3 ITLN1 gene-edited tracheal donors re-cultured and differentiated at ALI for MCM assays. Box plots show the log of average particle speed (b) or average total displacement (c) within control edited (scrb) and *ITLN1* KO mucociliary cultures that were mock- (BSA, white boxes) or IL-13-stimulated (grey boxes), with replicate experiments carried out using no wash, PBS wash, and PBS-DTT wash regimes. Average values are colored by donor. There are between 6 and 10 datapoints for each condition-donor pair (6-10 fields of view distributed across two different inserts per condition). Sample sizes per box plot, left to right are 24, 24, 27, 24, 21, 20, 21, 20, 20, 20, 20, 20. Above the plots are given the estimated percent recovery (and associated p-values) of particle displacement (a) or speed (b) in IL-13-stimulated cultures relative to BSA cultures when in an *ITLN1* KO epithelium compared to the control (scrb) epithelium. Two-sided p-values are based on linear mixed model t-tests, with a random effect for donor, and use Satterthwaite approximation of degrees of freedom. Box plots are as in (a).

Source data are provided as a Source Data file.

SFigure 4

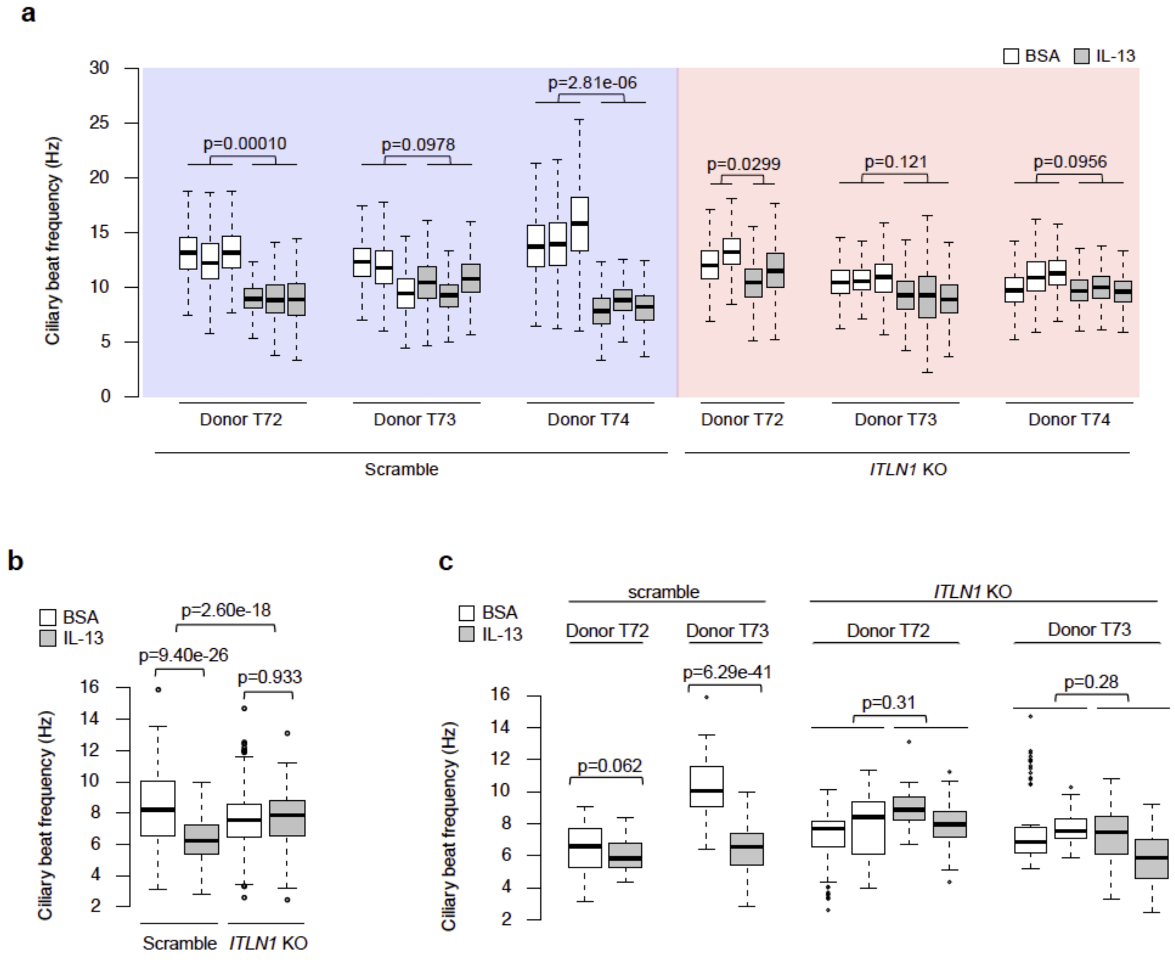
a
BSA
IL-13
Ciliary beat frequency (Hz)
30
25
20
15
10
5
0
p=0.00010
p=0.0978
p=2.81e-06
p=0.0299
p=0.121
p=0.0956
Donor T72
Donor T73
Donor T74
Donor T72
Donor T73
Donor T74
Scramble
ITLN1 KO
b
BSA
IL-13
p=2.60e-18
p=9.40e-26
p=0.933
Ciliary beat frequency (Hz)
16
14
12
10
8
6
4
2
Scramble
ITLN1 KO
c
scramble
ITLN1 KO
Donor T72
Donor T73
Donor T72
Donor T73
BSA
IL-13
p=6.29e-41
p=0.062
p=0.31
p=0.28
Ciliary beat frequency (Hz)
16
14
12
10
8
6
4
2

SFigure 4: ITLN-1 contributes to IL-13-mediated decrease in epithelial mucociliary function (ciliary beat frequency)

(a) Box plots of ciliary beat frequency (CBF) measured on control (scramble) and *ITLN1* KO mucociliary ALI cultures from triplicate culture inserts and 3 donors following mock- and IL-13-stimulation. Each box plot represents all datapoints collected from a given insert and donor (white = BSA, grey = IL-13). P-values are based on linear mixed model t-tests with random effects for donor and insert and use Satterthwaite approximation of degrees of freedom. Two-sided p-values directly above boxes are for tests of differences in CBF between BSA and IL-13. P-values at the top of the panel are for tests of donor-specific differences in the CBF response to IL-13 between scramble and *ITLN1* KO cultures. Box centers = median, upper and lower box bounds = 1st and 3rd quartiles, whiskers extend from these bounds up to 1.5 x IQR (inter-quartile range). All outliers have been omitted for visualization purposes. Number of CBF measures underlying each box are, from left to right, 618, 3,493, 3,404, 535, 2,929, 3,140, 3,716, 3,576, 2,327, 3,888, 3,775, 3,049, 5,656, 4,177, 3,932, 2,528, 2,715, 2,227, 4,175, 3,706, 3,359, 3,154, 2,075, 1,879, 1,955, 3,234, 3,234, 4,259, 3,872, 4,206, 3,665, 2,852, 2,215, 2,657.

(b) Independently performed ciliary beat frequency (CBF) experiment using the same 2 *ITLN1* gene-edited tracheal donors re-cultured and differentiated at ALI. Box plots of CBF are shown as measured on control (scramble) and *ITLN1* KO mucociliary ALI cultures treated with mock- and IL-13-stimulation. There was one culture insert for each treatment for scramble and two inserts per treatment for *ITLN1* KO. Each box plot represents all datapoints collected from all videos captured across donors and inserts for each condition (white = BSA, grey = IL-13). Two-sided p-values are based on linear mixed model t-tests with random effect for donor and use Satterthwaite approximation of degrees of freedom. Box plots are as in (a) except that data points beyond the whiskers are shown. Number of CBF measures underlying each box are, from left to right, 220, 150, 370, and 364.

(c) Box plots of CBF based on the validation experiment in b, where boxes are stratified by donor and insert. Two-sided p-values are based on mixed models implemented for scramble and *ITLN1* KO separately; for the *ITLN1* KO model, random effects were included for both donor and insert, whereas for the scramble model, a random effect was only included for donor. Box plots are as in (a) except that data points beyond the whiskers are shown. Number of CBF measures underlying each box are, from left to right, 110, 62, 110, 88, 92, 112, 92, 100, 73, 93, 102, and 70.

Source data are provided as a Source Data file.

SFigure 5

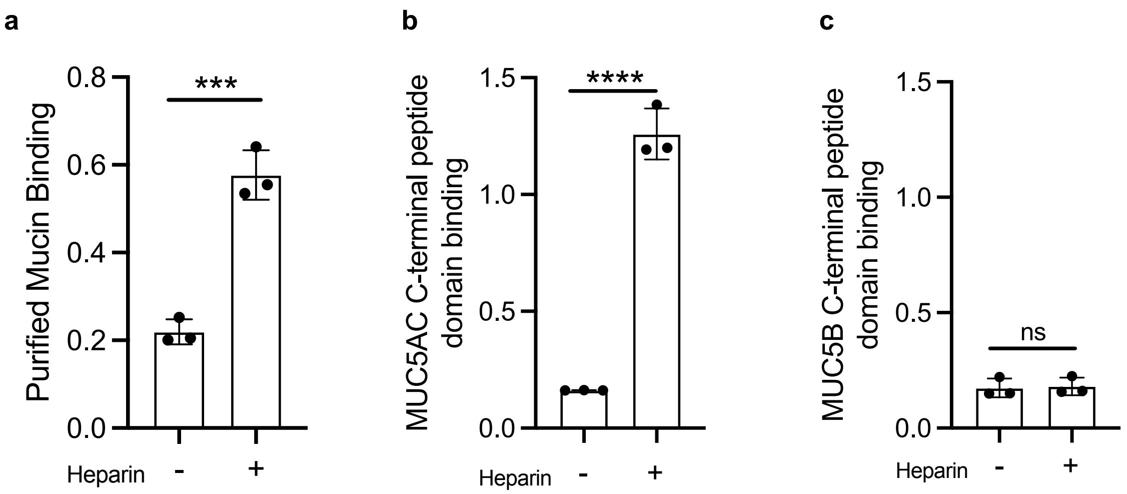

a
Purified Mucin Binding
0.8
0.6
0.4
0.2
0.0
***
Heparin
-
+
b
MUC5AC C-terminal peptide domain binding
1.5
1.0
0.5
0.0
****
Heparin
-
+
c
MUC5B C-terminal peptide domain binding
1.5
1.0
0.5
0.0
ns
Heparin
-
+

SFigure 5: Heparin binds to purified airway mucins and to the C-terminal domain of MUC5AC

(a) Bar graphs measuring binding of biotin-conjugated heparin to purified human airway mucins (isolated and pooled from n=5 participants). Data represents mean values +/- SD; Two-sided Student's T-test; *** p<0.001

(b) Bar graph measuring binding of biotin-conjugated heparin to the immobilized C-terminal domain peptides of MUC5AC. Data representative of n=3 independent experiments, Data represents mean values +/- SD; Two-sided Student's T-test; **** p<0.0001

(c) Bar graph measuring binding of biotin-conjugated heparin to the immobilized C-terminal domain peptides of MUC5B. Data representative of n=3 independent experiments, Data represents mean values +/- SD; Two-sided Student's T-test; ns=p-value not significant (p>0.05)

Source data are provided as a Source Data file.

SFigure 6

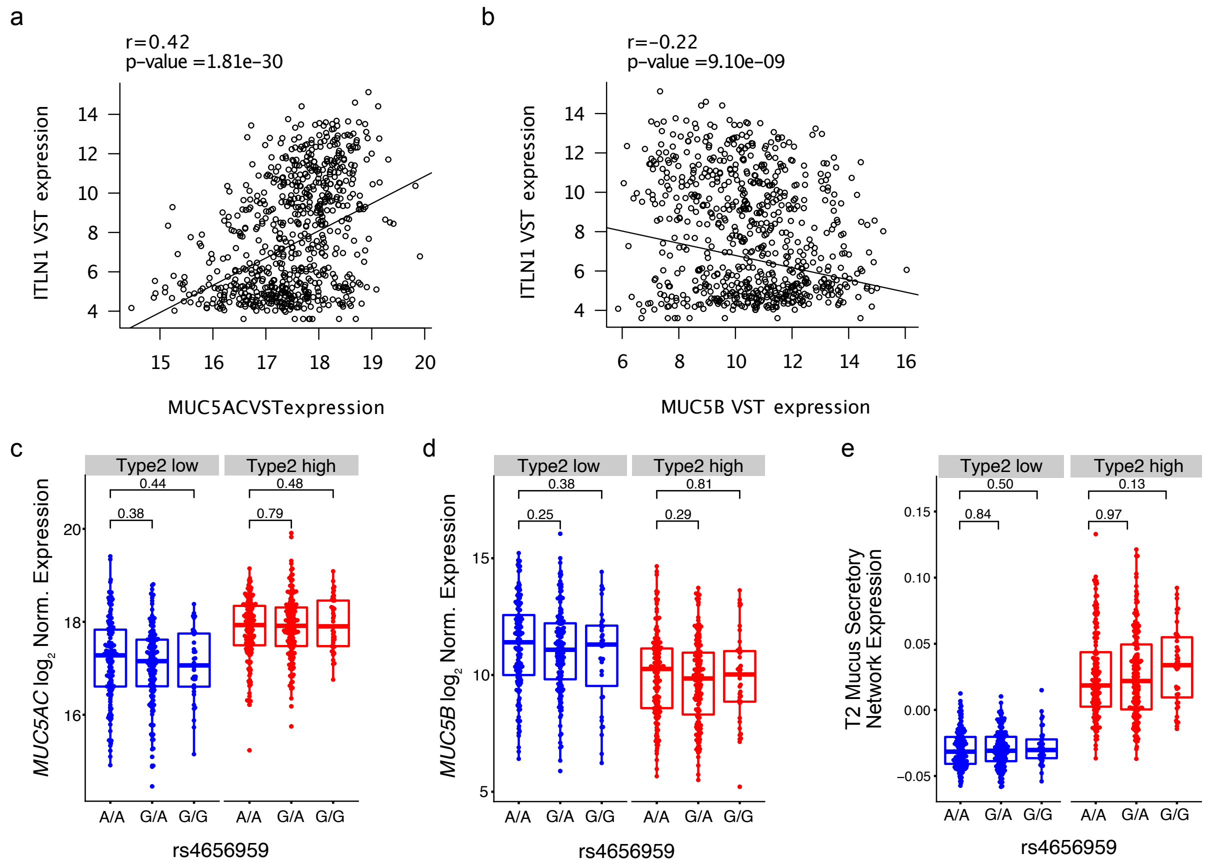

a
r=0.42
p−value =1.81e−30
ITLN1 VST expression
MUC5ACVSTexpression
b
r=−0.22
p−value =9.10e−09
ITLN1 VST expression
MUC5B VST expression
c
Type2 low
Type2 high
MUC5AC log2 Norm. Expression
rs4656959
d
Type2 low
Type2 high
MUC5B log2 Norm. Expression
rs4656959
e
Type2 low
Type2 high
T2 Mucus Secretory Network Expression
rs4656959

SFigure 6: Mucus metaplastic marker relationships with *ITLN1* expression and *ITLN1* genotype in GALA II nasal brushes

(a) Scatter plot of VST-normalized *ITLN1* and *MUC5AC* expression in nasal brushings from the GALA II study (n= 695). Pearson correlation coefficient and associated p-value are given.

(b) Scatter plot of VST-normalized *ITLN1* and *MUC5B* expression in nasal brushings from the GALA II study (n = 695). Pearson correlation coefficient and associated p-value are given.

(c) Box plots of log_2-normalized *MUC5AC* expression in nasal brushings from GALA II, stratified by T2 inflammation status and the *ITLN1* variant rs4656959 genotype (T2-low n: A/A=141, A/G=148, G/G=35 and T2-high n: A/A=151, A/G=165, G/G=41). Two-sided p-values were obtained using a Wilcox test. Box centers = median, upper and lower box bounds = 1st and 3rd quartiles, whiskers extend from these bounds up to 1.5 x IQR (inter-quartile range). All data points are overlain.

(d) Box plots of log_2-normalized *MUC5B* expression in nasal brushings from GALA II, stratified by T2 inflammation status and the *ITLN1* variant rs4656959 genotype (T2-low n: A/A=141, A/G=148, G/G=35 and T2-high n: A/A=151, A/G=165, G/G=41). Two-sided p-values were obtained using a Wilcox test. Box plots are as in (c).

(e) Box plots of the T2 mucus secretory network expression in the GALA II study stratified by T2 inflammation status and the *ITLN1* variant rs4656959 genotype (T2-low n: A/A=141, A/G=148, G/G=35 and T2-high n: A/A=151, A/G=165, G/G=41). Two-sided p-values were obtained using a Wilcox test. Box plots are as in (c).

Source data are provided as a Source Data file.

SFigure 7

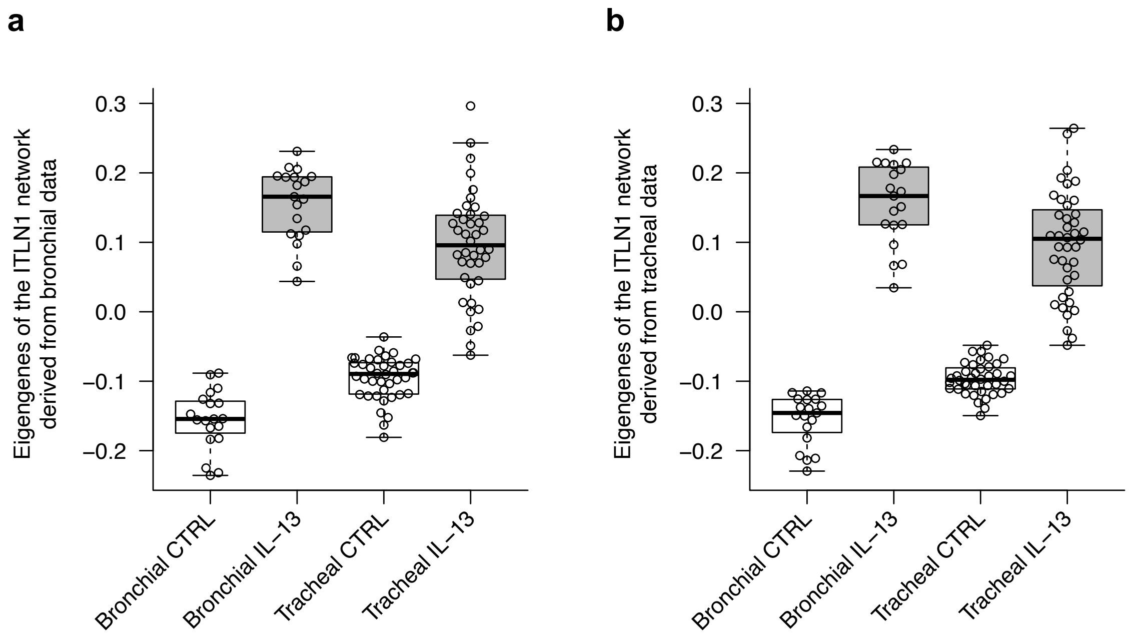
a
b
Eigengenes of the ITLN1 network
derived from bronchial data
Eigengenes of the ITLN1 network
derived from tracheal data
0.3
0.2
0.1
0.0
−0.1
−0.2
Bronchial CTRL
Bronchial IL−13
Tracheal CTRL
Tracheal IL−13

SFigure 7: Bronchial and tracheal-derived mucociliary epithelial cultures generate similar IL-13-stimulated *ITLN1*-containing T2 inflammatory networks

(a) Box plots showing eigengene expression of the *ITLN1*-containing T2 inflammatory bronchial network in control and IL-13 treated bronchial and tracheal culture expression data.

(b) Box plots showing eigengene expression of the *ITLN1*-containing T2 inflammatory tracheal network in control and IL-13-stimulated bronchial cultures in bronchial and tracheal culture expression data.

Box centers = median, upper and lower box bounds = 1st and 3rd quartiles, whiskers extend from these bounds up to 1.5 x IQR (inter-quartile range). All data points are overlain and data represents data analyzed from n=19 bronchial donors and n=40 tracheal donors.

Source data are provided as a Source Data file.

SFigure 8

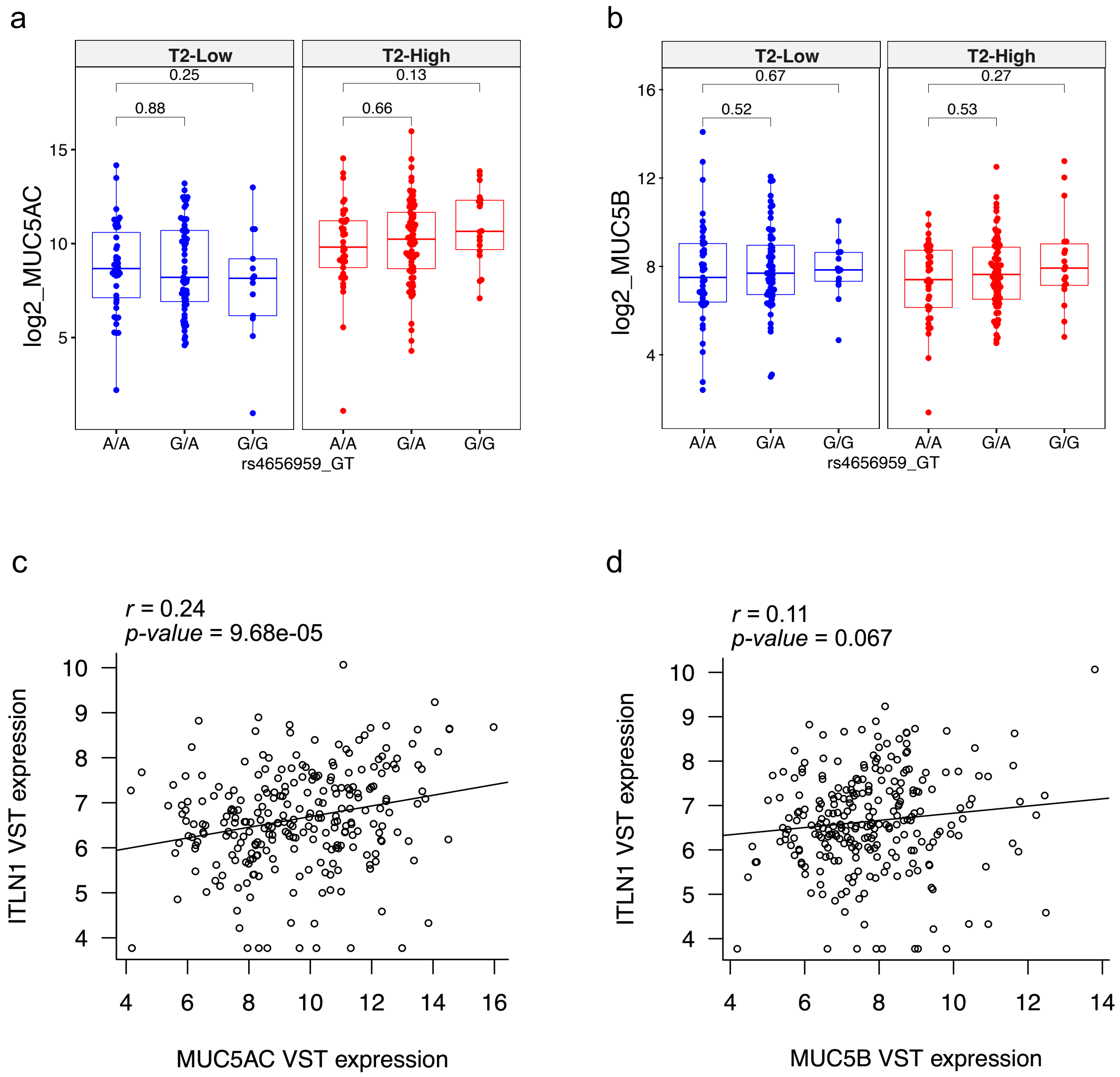
a
T2-Low
T2-High
0.25
0.88
0.13
0.66
log2_MUC5AC
15
10
5
A/A
G/A
G/G
rs4656959_GT
b
0.67
0.52
0.27
0.53
log2_MUC5B
16
12
8
4
c
r = 0.24
p-value = 9.68e-05
ITLN1 VST expression
MUC5AC VST expression
d
r = 0.11
p-value = 0.067
MUC5B VST expression

SFigure 8: Mucus metaplastic marker relationships with *ITLN1* expression and *ITLN1* genotype in SARP sputum

(a) Box plots of log_2-normalized *MUC5AC* expression in SARP sputum samples, stratified by T2 inflammation status and the *ITLN1* variant rs4656959 genotype (T2-low n: A/A=43, A/G=61, G/G=13 and T2-high n: A/A=37, A/G=77, G/G=18). Two-sided p-values were obtained using a Wilcox test. Box centers = median, upper and lower box bounds = 1st and 3rd quartiles, whiskers extend from these bounds up to 1.5 x IQR (inter-quartile range). All data points are overlain.

(b) Box plots of log_2-normalized *MUC5B* expression in SARP sputum samples, stratified by T2 inflammation status and the *ITLN1* variant rs4656959 genotype (T2-low n: A/A=43, A/G=61, G/G=13 and T2-high n: A/A=37, A/G=77, G/G=18). Two-sided p-values were obtained using a Wilcox test. Box plots are as in (a).

(c) Scatter plot of VST-normalized *ITLN1* and *MUC5AC* expression in sputum pellet samples from the SARP study (n= 260). Pearson correlation coefficient and associated p-value are given.

(d) Scatter plot of VST-normalized *ITLN1* and *MUC5B* expression in sputum pellet samples from the SARP study (n = 260). Pearson correlation coefficient and associated p-value are given.

Source data are provided as a Source Data file.
