# Supplementary material for: A common polymorphism in the Intelectin-1 gene influences mucus plugging in severe asthma
Source: Nat Commun. 2024 May 9;15:3900. doi: 10.1038/s41467-024-48034-5 (PMC11082194; doi:10.1038/s41467-024-48034-5)
Supplement: Supplementary file 2 — Description of Additional Supplementary Files [file 41467_2024_48034_MOESM2_ESM.pdf]

Description of Additional Supplementary Files

Supplementary Data Legends

Supplementary Data 1 ITLN1 network of 598 genes identified from WGCNA of IL-13-stimulated HBEC ALI cultures. ITLN1_cor gives the Spearman correlation coefficient between ITLN1 and the given network gene and pvalue_cor and qvalue_cor give the uncorrected and FDR-corrected (Benjamini-Hochberg method) two-sided p-values for this correlation, respectively. KME gives the module eigengene-based intramodular connectivity and kWithin gives non-eigengene-based intramodular connectivity. logFC_IL13 gives the log2 fold change of the gene in IL-13-stimulated versus control cultures and FDR_IL13 gives Benjamini-Hochberg-corrected two-sided p-values for this change based on a Wald test in DESeq2.

Supplementary Data 2 Bronchial epithelial brushing scRNA-seq cell type markers. For each marker, avg_log2FC gives the average log2 fold change of the marker in the cell type given in cell_type versus all other cells in the dataset. p_val and p_val_adj give uncorrected and FDR-corrected two-sided p-values for that change based on a Wilcox test using Seurat's FindMarkers function. pct.1 gives the proportion cell_type cells expressing the gene and pct.2 gives the proportion of non-cell_type cells expressing the gene.

Supplementary Data 3 Multivariable modeling of ITLN1 expression in nasal brushes collected from asthmatics in the GALA II asthma study. Model results are based on linear regression of normalized ITLN1 expression against age, sex, asthma status, type 2 status, genotype, and type 2 status x genotype interaction. The pvalues are two-sided and uncorrected and based on a t test.

Supplementary Data 4 T2 inflammation network genes from WGCNA performed on sputum gene expression from participants in the SARP study.

Supplementary Data 5 Summary table of demographic information from all participants who provided samples in each cohort analyzed, including age, sex, health status, and where applicable, T2-inflammation status and ITLN1 rs4656959 genotype.
