# Supplementary material for: A common polymorphism in the Intelectin-1 gene influences mucus plugging in severe asthma
Source: Nat Commun. 2024 May 9;15:3900. doi: 10.1038/s41467-024-48034-5 (PMC11082194; doi:10.1038/s41467-024-48034-5)
Supplement: Supplementary file 8 — Reporting Summary [file 41467_2024_48034_MOESM8_ESM.pdf]

nature portfolio

Corresponding author(s): Max A. Seibold
Last updated by author(s): Jan 19, 2024

Reporting Summary

Nature Portfolio wishes to improve the reproducibility of the work that we publish. This form provides structure for consistency and transparency in reporting. For further information on Nature Portfolio policies, see our Editorial Policies and the Editorial Policy Checklist.

Statistics

For all statistical analyses, confirm that the following items are present in the figure legend, table legend, main text, or Methods section.

n/a	Confirmed	
☐	☒	The exact sample size (*n*) for each experimental group/condition, given as a discrete number and unit of measurement
☐	☒	A statement on whether measurements were taken from distinct samples or whether the same sample was measured repeatedly
☐	☒	The statistical test(s) used AND whether they are one- or two-sided<br>*Only common tests should be described solely by name; describe more complex techniques in the Methods section.*
☐	☒	A description of all covariates tested
☐	☒	A description of any assumptions or corrections, such as tests of normality and adjustment for multiple comparisons
☐	☒	A full description of the statistical parameters including central tendency (e.g. means) or other basic estimates (e.g. regression coefficient) AND variation (e.g. standard deviation) or associated estimates of uncertainty (e.g. confidence intervals)
☐	☒	For null hypothesis testing, the test statistic (e.g. *F*, *t*, *r*) with confidence intervals, effect sizes, degrees of freedom and *P* value noted<br>*Give P values as exact values whenever suitable.*
☒	☐	For Bayesian analysis, information on the choice of priors and Markov chain Monte Carlo settings
☒	☐	For hierarchical and complex designs, identification of the appropriate level for tests and full reporting of outcomes
☐	☒	Estimates of effect sizes (e.g. Cohen's *d*, Pearson's *r*), indicating how they were calculated

Our web collection on statistics for biologists contains articles on many of the points above.

Software and code

Policy information about availability of computer code

Data collection

None

Data analysis

Revolve R4 EchoPro app v6.4.1 for IHC and IF image capture;
QuickTime v10.5 was used for particle tracking video acquisition;
Zeiss ZEN Blue Lite was used for confocal image analysis
Skewer version 0.2.2 was used for trimming bulk RNA-seq data;
GSNAP version 2016_05_01 was used for mapping bulk RNA-seq data from GALA II;
HISAT2 version 2.1.0 was used for mapping bulk RNA-seq data from ALI cultures;
HTSeq-count version 0.9.1 was used for bulk-RNA seq data gene quantification;
DESeq2 version 1.22.2 was used to normalize bulk RNA-seq expression data and differential expression;
WGCNA version 1.68 was used to perform network analysis;
DataAnalysis version 4.2 was used for mass spectrometry (MS) analysis;
Mascot version 2.4 was used for peptide identification after MS;
ProteinScape version 3.0 was used for filtering and assessing peptides from MS;
edgeR version 3.28.0 was used for differential abundance of peptides and for TMM normalization;
ImageJ v1.53a was used for western blot imaging;
trackpy version 0.4.1 was used for particle tracking analysis;
python version 3.6.2 was used for particle tracking analysis;
lmerTest version 3.1.3 was used for running linear mixed models;
Cell Ranger version 4 was used for processing scRNA-seq data;
Seurat v4.0 was used for analyzing scRNA-seq data;

SAS version 9.04 was used for analysis for mucus plug score analysis;
ADMIXTURE version 1.3 was used on genotype data to compute admixture factors;
PEER version 1.3 was used to generate PEER factors;
FastQTL version 2.184 was used to perform cis-eQTL analysis;
QTLTools version 1.1 was used to perform stepwise regression;
enrichR version 3.0 was used for functional enrichment analysis;
R version 3.4.1 was used for analysis and visualization;
CIBERSORTx was used for cell type deconvolution
CRISPRdesign v1 tool was used to chose gRNA sequences for gene editing;
ICE Analysis Tool v3.0 was used for Inference of CRISPR Edits (ICE) analysis

For manuscripts utilizing custom algorithms or software that are central to the research but not yet described in published literature, software must be made available to editors and reviewers. We strongly encourage code deposition in a community repository (e.g. GitHub). See the Nature Portfolio guidelines for submitting code & software for further information.

Data

Policy information about availability of data

All manuscripts must include a data availability statement. This statement should provide the following information, where applicable:

- Accession codes, unique identifiers, or web links for publicly available datasets
- A description of any restrictions on data availability
- For clinical datasets or third party data, please ensure that the statement adheres to our policy

Provided Data Availability and Code Availability Statement provided in the manuscript:
Data Availability
Gene lists associated with main Figure and Supplementary Figure panels can be found in the Supplementary Tables provided with this manuscript. GALA II RNA-seq data used in this study has been previously deposited in the National Center for Biotechnology Information/Gene Expression Omnibus (GEO) GSE152004 [https://www.ncbi.nlm.nih.gov/geo/query/acc.cgi?acc=GSE152004]. Data used as part of the Human Lung Atlas for scRNAseq analyses can be found in the Supplementary Data and at https://hlca.ds.czbiohub.org/ as reported in the cited manuscript by Travaglini, et al. The raw and processed scRNA-seq data used in this study have been deposited in the National Center for Biotechnology Information/Gene Expression Omnibus (GEO) with accession number GSE254127 [https://www.ncbi.nlm.nih.gov/geo/query/acc.cgi?acc=GSE254127]. The processed protein and RNA-seq gene expression data generated from in vitro experiments can be found at the github repository [https://github.com/seiboldlab/ITLN1_paper]. Data underlying manuscript figures involving the SARP cohort are provided in the github repository. Source data are provided with this paper.

Code Availability Statement
The code used to produce figures and perform analyses in the study can be found on GitHub [https://github.com/seiboldlab/ITLN1_paper].

Additional details if desired:
TOPMed freeze 8 variant calls are available from dbGaP accession phs000920.v2.p2 [https://www.ncbi.nlm.nih.gov/projects/gap/cgi-bin/study.cgi?study_id=phs000920.v4.p2];

Enrichr libraries used:
- GO Biological Process 2018 table [https://maayanlab.cloud/Enrichr/geneSetLibrary?mode=text&libraryName=GO_Biological_Process_2018];
- GO Molecular Function 2018 table [https://maayanlab.cloud/Enrichr/geneSetLibrary?mode=text&libraryName=GO_Molecular_Function_2018];
- GO Cellular Component 2018 table [https://maayanlab.cloud/Enrichr/geneSetLibrary?mode=text&libraryName=GO_Cellular_Component_2018];
- Ligand Perturbations from GEO up table [https://maayanlab.cloud/Enrichr/geneSetLibrary?mode=text&libraryName=Ligand_Perturbations_from_GEO_up];
- Kyoto Encyclopedia of Genes and Genomes 2019 Human table [https://maayanlab.cloud/Enrichr/geneSetLibrary?mode=text&libraryName=KEGG_2019_Human];
- Reactome 2016 table [https://maayanlab.cloud/Enrichr/geneSetLibrary?mode=text&libraryName=Reactome_2016];

Research involving human participants, their data, or biological material

Policy information about studies with human participants or human data. See also policy information about sex, gender (identity/presentation), and sexual orientation and race, ethnicity and racism.

Reporting on sex and gender	We used self-reported gender as covariate in the ITLN1 eQTL study of GALA II and SARP. In the GALA II study, sex check is performed by TOPMED to match the self-reported gender to genotyped determined sex.
Reporting on race, ethnicity, or other socially relevant groupings	*Please specify the socially constructed or socially relevant categorization variable(s) used in your manuscript and explain why they were used. Please note that such variables should not be used as proxies for other socially constructed/relevant variables (for example, race or ethnicity should not be used as a proxy for socioeconomic status).*<br>*Provide clear definitions of the relevant terms used, how they were provided (by the participants/respondents, the researchers, or third parties), and the method(s) used to classify people into the different categories (e.g. self-report, census or administrative data, social media data, etc.)*<br>*Please provide details about how you controlled for confounding variables in your analyses.*
Population characteristics	The Genes-Environment & Admixture in Latino Americans study (GALA II) is an on-going case-control study of asthma in Latino children and adolescents. The study includes subjects with asthma and healthy controls of Latino descent between the ages of 8 and 21.<br><br>The Severe Asthma Research Program (SARP III) is an on-going longitudinal characterization study of asthma. The study to

date includes adults (>18 years old).

The Epithelial Barriers in Asthma, Eczema, Food Allergy, and GERD study is an on-going characterization study of pediatric asthma. The study to date includes pediatric participants (<18 years old).

Tracheal specimens were collected as part of NJH and UCSF institutional tissue bank protocols.

Recruitment

GALA II Study subjects were recruited from the community centers and clinics in the mainland U.S. and Puerto Rico (2006-present). Asthma case status was physician-diagnosed. Recruited subjects completed in-person questionnaires detailing medical, environmental, and demographic information.

SARP III study subjects were recruited from community clinics and hospitals in the United States. Asthma case status was physician-diagnosed. Recruited subjects completed in-person questionnaires detailing medical, environmental, and demographic information.

Participants in the Epithelial Barriers in Asthma, Eczema, Food Allergy, and GERD study are recruited at National Jewish Health and asthma case status was physician-diagnosed.

We are not aware of any biases in our recruitment that would adversely affect our results.

Ethics oversight

All research conducted in these studies complies with all relevant ethical regulations. Human bronchial and tracheal airway epithelial cells from adult subjects were banked in a de-identified fashion and obtained for research purposes from the National Jewish Health (NJH) Live Cell Core. The NJH Live Cell Core is an institutional review board-approved study (HS-2240) for the collection of tissues from consented patients for researchers at NJH. For tracheal epithelial cell isolation, human lung specimens were obtained from de-identified lung donors whose lungs were not suitable for transplantation from the International Institute for the Advancement of Medicine (Edison, NJ), and Donor Alliance of Colorado. Written consent for research on tracheal and lung specimens was obtained by the Organ Procurement Organization prior to organ donation. The National Jewish Health Institutional Review Board (IRB) approved the research on lung cells under IRB protocol HS-3209. Nasal airway epithelial brushes were collected from subjects recruited as part of the Genes-environments and Admixture in Latino Americans II (GALA II) childhood asthma study to be used in genome-wide genetic and genomic analysis,, which was approved by local institutional review boards (UCSF, IRB number 10–00889, Reference number 153543, NJH HS-2627). All subjects and their parents provided written informed assent and written informed consent for sample collection and use of the samples in genetic and genomic analyses30,31. Bronchial airway brushings used for scRNAseq were collected following informed consent and assent, as part of the Epithelial Barriers in Asthma, Eczema, Food Allergy, and GERD study approved by the BRANY IRB for National Jewish Health under protocol HS-3255-528. Airway tracheal epithelial cells used to model the IL-13 reponse compared to bronchial responses were harvested following appropriate consent for research from the tracheas of organ donors obtained from the California Transplant Donor Network32. Sputum specimens and mucus plug scores were collected following informed consent from participants, into the Severe Asthma Research Program (SARP 3) study, and the study was approved by the IRBs at all participating centers: Brigham and Women's Hospital (Partners Human Research Committee, 2012P001528), Boston Children's Hospital (Boston Children's Hospital IRB, IRB-P00004759), University of Wisconsin (University of Wisconsin Madison Health Sciences IRB, 2012-0571-CP002), University of Pittsburgh (Univeristy of Pitsburgh IRB, PRO12070359), Washington University (Washington University in St. Louis IRB, 201206102), University of California – San Francisco (University of California – San Francisco IRB, 12-09392; 12-09556), University of Virginia (IRB for Health Sciences, 16400), Clevland Clinic (Clevland Clinic IRB, 6185), Virginia Commonwealth Univeristy (Virginia Commonwealth University IRB, HM14883), Rainbow Babies (University Hospital IRB, 09-12-08), Wake Forest (Wake Forest IRB, IRB00021507), Emory University (Emory University IRB, IRB00058103).

Note that full information on the approval of the study protocol must also be provided in the manuscript.

Field-specific reporting

Please select the one below that is the best fit for your research. If you are not sure, read the appropriate sections before making your selection.

☒ Life sciences ☐ Behavioural & social sciences ☐ Ecological, evolutionary & environmental sciences

For a reference copy of the document with all sections, see nature.com/documents/nr-reporting-summary-flat.pdf

Life sciences study design

All studies must disclose on these points even when the disclosure is negative.

Sample size

For GALA II nasal transcriptomic study, no formal sample size calculation was done. The analysis from from this dataset was conducted on all participants with RNA-seq data (n=695) and was used to describe network associations with ITLN1 expression, and to interrogate ITLN1 expression among subjects stratified by Type 2 inflammation. Approximately 50% of the GALA II participants present with T2 inflammation, which provides a sufficient number of participants to analyze ITLN1 expression across T2low and T2high endotype groups.

For GALA II eQTL study, no formal sample size calculation was done. There are 681 donors with paired genotype and expression data which is larger than the sample size of any single tissue in GTEx.

For the analysis of RNA-seq expression data and genotype-specific effects, and aqueous and mucus fraction proteomics of HBEC ALI cultures between paired IL13-stimulated and control samples, no formal sample size calculation was done. This sample size (n=19, 14, and 9, respectively) included at least n=3 participants per genotype group, which is sufficient to identify ITLN1 expression changes following IL-13 treatment.

For the KO of ITLN-1 to determine its role in mucociliary functions in tracheal ALI cultures between paired IL13-stimulated and control samples, no formal sample size calculation was done. This sample size (n=3) was sufficient to identify robust KO of ITLN-1 following IL-13 treatment and effects on mucociliary movement. For capture of cilia beat frequency analysis, the sample size of 6 videos per insert, performed in triplicate culture inserts (n=3) per donor per treatment group was sufficient to identify changes in cilia beat frequency by gene editing and treatment conditions.

For the analysis of ITLN-1 mucin binding assay data, no formal sample size calculation was done. This sample size (n=3) was sufficient to identify changes in ITLN-1 binding capacity to purified mucins, and specific mucin domains.

For the analysis of ITLN1 gene and protein expression and secretion in tracheal ALI cultures between paired IL13-stimulated and control samples, no formal sample size calculation was done. This sample size (rs4656959 [G/G] n=5 and rs4656959 [A/A]) was sufficient to identify ITLN1 gene and protein expression changes, and apical secretion by genotype and treatment.

For the SARP RNAseq gene expression analysis and mucus plug score analysis, no sample size calculation was performed. This dataset was used to analyze ITLN1 expression in sputum samples from subjects stratified by type 2 inflammation status, and to analyze association of mucus plug scores with ITLN rs4656959 genotype. There are 249 subjects in the RNAseq data set and 112 subjects with paired mucus plug scores that were used for this analysis.

For bronchial brush scRNA-seq data, the sample size is 2. This data is used to identify the epithelial cell type(s) expressing ITLN1. Our result is consistent with the result from the human lung cell atlas and the Tabula Sapiens. Utilization of a total of 11,515 cells for analysis generated 17 distinct cell clusters which results in a sufficient number of cells per group to determine cell type expression patterns of ITLN1 in the bronchial brushings.

Data exclusions

For GALA II eQTL study, we excluded 14 donors that failed genotyping QC from TOPMED.

Replication

For GALA II eQTL study, no replication was performed, but results are robust due to the large sample size. There are 681 donors with paired genotype and expression data which is larger than the sample size of any single tissue in GTEx. Replication was not performed as for the eQTL study, however Identification of ITLN1 eQTL reported in this manuscript and the effect of the ITLN1 variant on ITLN1 gene expression and protein expression of ITLN-1 has been validated in both bronchial and tracheal-derived airway samples in vitro.

For immunofluorescence analysis on histology sections we captured 6-10 fields of view per condition over 8 total donor cultures. Labeling results have been independently replicated successfully across all donor samples.

For proteomics, apical aqueous and mucus fractions from duplicate (n=2) culture inserts per donor were collected and pooled for analysis across 14 donors for the aqueous fraction and 9 donors for the mucus fraction. Mucus fraction ITLN-1 measurements were successfully replicated from all cultured donors following the the re-derivation of ALI cultures in an independent experiment.

For confocal analysis, 10 fields of view were captured by z-stack image capture and used for analysis from 3 donors

Samples for KO analysis were harvested from 3 culture inserts per donor and pooled for HRM DNA editing and Western Blot analysis
For primary MCM analyses, 10sec videos were captured at 6 fields of view per insert on triplicate culture inserts per treatment per donor (n=3 donors) and used for analysis. Experiment was independently repeated using 10sec videos captured at 4 fields of view per insert on duplicate culture inserts per treatment per donor and used for analysis.

For CBF assays, primary experiments utilized triplicate culture inserts per treatment were used to capture CBF videos from scrb control:no IL13 = 30,899; scrb control:IL-13 = 24,786, ITLN1 KO:no IL-13 = 25,533, ITLN1 KO:IL-13 = 24,964 fields of view, and were used for analysis. An independently replicated experiment was performed and utilized a single culture insert for scrb conditions and duplicate culture inserts for KO conditions that were used to capture CBF videos from scrb control:no IL13 = 220; scrb control:IL-13 = 150, ITLN1 KO:no IL-13 = 370, ITLN1 KO:IL-13 = 364 fields of view, and were used for analysis.

For mucin binding assays, airway mucins were purified from n=5 subjects and pooled for binding assays, and all assay conditions were performed in triplicate.

For single Cell RNAseq study, no replication was performed. However, our results largely mirrored the results from the human lung cell atlas and the Tabula Sapiens.

For tracheal genotype experiments, all samples for qPCR and Western blot assays were collected and pooled from triplicate culture inserts for analyses, qPCR assays were run in duplicate per treatment condition.

Randomization

Randomization was not applicable to eQTL analysis as there were no experimental groups. The eQTL analysis was adjusted age, sex, bmi, asthma status, and admixtures estimates to account for confounders.

For the bronchial analysis of ITLN1 RNA-seq expression and protein secretion data, we performed stratified sampling by randomly select 19 HBEC donors from the NJH Biobank. For analysis of ITLN1 expression by health status, asthma was physician defined in the biobank database, and healthy controls were defined by the lack of asthma diagnosis or other confounding airway disease (CF, COPD, active infection, etc). Genotype groups were defined by data obtained by genotype assay for ITLN1 rs4656959 GG, GA, or AA.

For the tracheal analysis of ITLN1 RNA-seq and protein expression data, we performed stratified sampling by randomly selecting five tracheal airway donors with rs4656959-GG and five tracheal airway donors with rs4656959-AA genotypes.

Randomization was not applicable to the SARP mucus plugging score analysis as there were no experimental groups. Analysis was adjusted for age, sex, and admixtures estimates to account for confounders.

Blinding

All proteomic samples, histological sections used for immunofluorescence, samples used for Western Blot analysis, and images/videos

Blinding

analyzed for MCM particle tracking and CBF were collected and labeled in a blinded fashion with de-identified IDs prior to analyses.

For the experiments using an ALI mucociliary culture system, we employed a paired experiment design and as a result blinding is not required.

For eQTL analysis, we conducted an association between rs4656959 genotype groups and ITLN1 expression under additive genetic model. Since we are not recruiting subjects based on their genotype of rs4656959, blinding was unnecessary.

Reporting for specific materials, systems and methods

We require information from authors about some types of materials, experimental systems and methods used in many studies. Here, indicate whether each material, system or method listed is relevant to your study. If you are not sure if a list item applies to your research, read the appropriate section before selecting a response.

Materials & experimental systems

n/a	Involved in the study
☐	☒ Antibodies
☐	☒ Eukaryotic cell lines
☒	☐ Palaeontology and archaeology
☒	☐ Animals and other organisms
☒	☐ Clinical data
☒	☐ Dual use research of concern
☒	☐ Plants

Methods

n/a	Involved in the study
☒	☐ ChIP-seq
☒	☐ Flow cytometry
☒	☐ MRI-based neuroimaging

Antibodies

Antibodies used

Rabbit anti-human Intelectin-1 (ITLN-1) Abcam ab118232 (IF and WB 1:200), Mouse anti-human MUC5AC Fisher Scientific MA1-21907 (IF 1:500; Dot Blot 1:1000), Rabbit anti-human MUC5B Santa Cruz sc20119 (1:200), AlexaFluor488 goat anti-rabbit IgG (1:500, ThermoScientific, A-11008), AlexaFluor594 goat anti-mouse IgG (1:500, ThermoScientific, A-11005), HRP-conjugated goat anti-rabbit (1:10,000; BioRad #170-6515), HRP-conjugated goat anti-mouse (1:10,000; BioRad #170-6516)

Validation

MUC5AC antibody underwent Advanced Verification as stated on the Thermo website with over 210 published figures and references (https://www.thermofisher.com/antibody/product/MUC5AC-Antibody-clone-45M1-Monoclonal/MA1-21907). Intelectin-1 antibody was validated across several applicable tissue types, and sequence target references to several protein sequence databases (https://www.abcam.com/itln1-antibody-ab118232.html). MUC5B antibody underwent several round of validation with various published references and citations (https://www.scbt.com/p/mucin-5b-antibody-h-300)

Eukaryotic cell lines

Policy information about cell lines and Sex and Gender in Research

Cell line source(s)

NIH 3T3 Mouse Fibroblast cells were purchased from ATCC (CRL-1658) for use as feeder co-culture for primary human epithelial cells.

Primary cell lines cultured from 13 tracheal donors for ITLN1 genotype expression confirmation assays were from 5 female and 8 male participants. Cells cultured from bronchial brushes were from 11 female and 9 male participants. Tracheal cells used for IL13 expression network analysis were cultured from 20 male, 12 female, and 8 NA participants. Complete demographics for all donors can be found as listed in the provided Data Availability statement

Authentication

No authentication was performed

Mycoplasma contamination

No mycoplasma testing was performed

Commonly misidentified lines (See ICLAC register)

No commonly misidentified lines were used

Plants

Seed stocks	NA
Novel plant genotypes	NA
Authentication	NA
